# Modelling the impact of increased alcohol taxation on alcohol-attributable cancers in the WHO European Region

**DOI:** 10.1016/j.lanepe.2021.100225

**Published:** 2021-09-15

**Authors:** Carolin Kilian, Pol Rovira, Maria Neufeld, Carina Ferreira-Borges, Harriet Rumgay, Isabelle Soerjomataram, Jürgen Rehm

**Affiliations:** a Institute of Clinical Psychology and Psychotherapy, Technische Universität Dresden, Dresden, Germany; b Program on Substance Abuse, Public Health Agency of Catalonia, Barcelona, Spain; c WHO European Office for Prevention and Control of Noncommunicable Diseases, Moscow, Russian Federation; d Institute for Mental Health Policy Research, Centre for Addiction and Mental Health (CAMH), Toronto, ON, Canada; e Cancer Surveillance Branch, International Agency for Research on Cancer, Lyon, France; f Dalla Lana School of Public Health, University of Toronto, Toronto, ON, Canada; g Campbell Family Mental Health Research Institute, CAMH, Toronto, ON, Canada; h Department of Psychiatry, University of Toronto, Toronto, ON, Canada; i Department of International Health Projects, Institute for Leadership and Health Management, I.M. Sechenov First Moscow State Medical University, Moscow, Russian Federation; j Centre for Interdisciplinary Addiction Research, University Medical Center Hamburg-Eppendorf, Department of Psychiatry, Hamburg, Germany

**Keywords:** Alcohol, cancer, alcohol-attributable cancer, taxation, WHO European Region

## Abstract

**Background:**

Reducing the alcohol-attributable cancer burden in the WHO European Region is a public health priority. This study aims to estimate the number of potentially avoidable cancers in countries of the WHO European Region in 2019 for three scenarios in which current excise duties on alcoholic beverages were increased by 20%, 50%, or 100%.

**Methods:**

Mean prices and excise duties for beer, wine, and spirits in the Member States of the WHO European Region in 2020 were used as the baseline scenario. We assumed that increases in excise duties (20%, 50%, and 100%) were fully incorporated into the consumer price. Beverage-specific price elasticities of demand, with lower elasticities for heavy drinkers, were obtained from a meta-analysis. Model estimates were applied to alcohol exposure data for 2009 and cancer incidence and mortality rates for 2019, assuming a 10-year lag time between alcohol intake and cancer development and mortality.

**Findings:**

Of 180,887 (95% Confidence interval [CI]: 160,595-201,705) new alcohol-attributable cancer cases and 85,130 (95% CI: 74,920-95,523) deaths in the WHO European Region in 2019, 5·9% (95% CI: 5·6-6·4) and 5·7% (95% CI: 5·4-6·1), respectively, could have been avoided by increasing excise duties by 100%. According to our model, alcohol-attributable female breast cancer and colorectal cancer contributed most to the avoidable cases and deaths.

**Interpretation:**

Doubling current alcohol excise duties could avoid just under 6% (or 10,700 cases and 4,850 deaths) of new alcohol-attributable cancers within the WHO European Region, particularly in Member States of the European Union where excise duties are in many cases very low.

**Funding:**

None.

**Research in context**

**Evidence before this study**

Alcohol is classified as Group 1 carcinogen, causally linked to seven different types of cancers. About 4·2% of the 4·8 million cancer cases in the WHO European Region in 2020 were estimated to be caused by alcohol. Increasing alcohol excise duties is one of the WHO's “Best Buys” to cost-effectively reduce alcohol use and the alcohol-attributable health burden, yet it is the least implemented alcohol control policy in the Region. Prior modelling studies showed that a 100% increase of national alcohol excise duties in four countries of the WHO European Region with differing levels of alcohol use and alcohol policies could prevent up to 7% of new alcohol-attributable cancer cases in these countries.

**Added value of this study**

This study models the impact of higher alcohol excise duties on the alcohol-attributable cancer burden for countries of the WHO European Region. Three different taxation increase scenarios were modelled, increasing 2020 excise duties by 20%, 50% or 100%. Within the Region, 5·9% (95% Confidence Interval [CI]: 5·6-6·4) of new alcohol-attributable cancer cases and 5·7% (95% CI: 5·4-6·1) of alcohol-attributable cancer deaths could have been avoided in 2019 if excise duties were increased by 100%, with female breast cancer and colorectal cancer demonstrating the greatest reductions. For smaller tax increases, a relatively lower percentage of new alcohol-attributable cancer cases and deaths could have been avoided.

**Implications of all the available evidence**

Our modelling study indicates that increasing excise duties on alcoholic beverages likely reduces the alcohol-attributable cancer burden in the WHO European Region. Given the prevailing low levels of taxation in many countries, particularly within the European Union, increasing excise duties represents a considerable and as yet untapped potential to tackle the alcohol-attributable cancer burden in the Region.

Alt-text: Unlabelled box

## 1 Introduction

Reducing the health burden of cancer is a major priority in the WHO European Region, as highlighted in the WHO's European Programme of Work, 2020-2025 [1] and in the recent pan-European movement, United Action Against Cancer, launched by the WHO with a vision to eliminate cancer as a life threatening disease [2]. Alcohol is a causal and modifiable risk factor for cancer [3], [4], [5]. In 2020, almost 4·8 million people in the WHO European Region developed cancer [6], of which an estimated 4·2% were causally linked to alcohol [7]. The International Agency for Research on Cancer classified alcohol as a Group 1 carcinogen [8], and in a comparison with several other key behavioural and environmental risk factors for cancer, alcohol was identified as the second leading cause after tobacco smoking for cancer development in France, a country with one of the largest populations of the WHO European Region [9]. The importance of alcohol as a risk factor for cancer and its prevention has been further stressed in a joint work of the WHO and the International Agency for Research on Cancer by publishing a factsheet on alcohol and cancer, appealing for better prevention and increased levels of political commitment [10].

The primary carcinogenic compound in alcoholic beverages is ethanol, whose product of metabolism acetaldehyde can lead to DNA damage [3,4]. Yet, there are also other mechanisms rendering ethanol a carcinogen: alterations in hormone levels, oxidative stress from chronic alcohol intake, and folate deficiency, which in turn can lead to impaired DNA methylation. These biological pathways already suggest that alcohol-attributable cancer is not only a concern of chronic and at-risk drinking, as any alcohol intake contributes to cancer development, albeit in a dose-response manner [11,12]. Accordingly, a recent study estimated that almost 23,000 new cancer cases in the European Union (EU), equivalent to 13·3% of all alcohol-attributable cancer cases in 2017, were due to moderate consumption (< 20 grams of pure alcohol per day or up to half a litre of beer) [13].

While the causal link between alcohol and cancer has been known for decades, sufficient action to reduce alcohol consumption has not been taken [10,14]. Cost-effective alcohol control policies, such as the WHO “Best Buys” [15], exist which can reduce the alcohol-attributable burden [16]. Among these policy options, increasing excise taxation on alcoholic beverages is one of the most promising measures to target the alcohol-attributable cancer burden, while being the least implemented policy in the Region to date [15]. For example, 23 of the 53 Member States in the WHO European Region, most of them in the EU, have no excise duty on wine [17], even though wine accounts for about one third in *per capita* consumption in the Region [14]. In an explorative modelling study, researchers have shown that up to 7% of cancer cases could potentially be avoided when raising the current excise duties on alcoholic beverages by 100% in four countries of the WHO European Region that have different levels of alcohol use as well as alcohol control policies (for a separate study on Germany, see [18]) [19].

This study estimates the number of new cancer cases and deaths that could have been averted in the WHO European Region in 2019 by applying different scenarios of tax increases to current national beverage-specific alcohol excise duties. Following the modelling approach of Rovira et al. [19], we modelled the effect of a 20%, 50% and 100% increase in excise taxes on new alcohol-attributable cancer cases and deaths.

## 2 Methods

This study adheres to the Guidelines for Accurate and Transparent Health Estimates Reporting (GATHER) statement (see Supplementary Table S1).

### 2.1 Sources of data

In order to analyse the impact of the different taxation increase scenarios, we first identified current taxation information and average alcoholic beverage prices for each country. For Member States of the EU, taxation information were available from the European Commission [20]. For the remaining countries, we relied on national data sources (for a complete list, see Supplementary Table S2). Average prices for three types of alcoholic beverages (beer, wine, and spirits) were obtained from the Statista webpage [21], the OECD Consumption Tax Trend [22], or from national sources (see Supplementary Table S2). Pricing information was transformed into international dollars (Int$) using the 2020 purchasing power parity conversion factor (GDP PPP) from the World Bank [23]. Data were extracted by two independent researchers and cross-checked with published data [17,24]. For all countries, we assumed a mean percentage of pure alcohol of 5%, 12·5%, and 40% for beer, wine, and spirits, respectively (similar assumptions as in [14]), and a gravity of 12 °Plato for beer, which is a measure used specifically for brewed beverages and describes the amount of dissolved solids from the malt and hops in water before fermentation. An overview of the current excise duties by beverage type and country is presented in Table 1. Data were available for all Member States, except Andorra, Monaco, and San Marino.

**Table 1:** Tax structure, mean price and share of tax rate in mean price per alcoholic beverage for Member States of the WHO European Region.

Country	Beer			Wine			Spirits		
	Tax structure	Mean price (Int$ / L)	% Tax	Tax structure	Mean price (Int$ / L)	% Tax	Tax structure	Mean price (Int$ / L)	% Tax
Albania	°alcohol	6·50	13·5	finished product	29·78	2·5	pure alcohol	29·36	21·8
Armenia	ad valorem tax	7·01	23·1	ad valorem tax	15·77	9·1	ad valorem tax	21·63	52·4
Austria	°plato	4·51	7·0	no excise duty	14·82	0·0	pure alcohol	36·30	17·4
Azerbaijan	finished product	6·94	10·6	finished product	18·51	2·0	finished product	27·54	21·4
Belarus	finished product	5·08	10·5	finished product	16·60	8·2	pure alcohol	23·60	36·2
Belgium	°plato	4·09	7·8	finished product	22·19	4·5	pure alcohol	48·02	33·0
Bosnian-Herzegovina	finished product	7·21	4·1	finished product	32·10	1·2	pure alcohol	37·51	23·8
Bulgaria	°plato	2·91	8·9	no excise duty	19·57	0·0	pure alcohol	21·60	29·4
Croatia	°alcohol	5·56	11·0	no excise duty	10·40	0·0	pure alcohol	26·99	23·9
Cyprus	°alcohol	7·31	6·8	no excise duty	28·31	0·0	pure alcohol	43·66	14·5
Czechia	°plato	3·46	8·9	no excise duty	9·23	0·0	pure alcohol	27·64	37·5
Denmark	°alcohol	4·66	7·8	finished product	29·21	7·7	pure alcohol	33·79	26·6
Estonia	°alcohol	3·45	33·8	finished product	19·80	13·7	pure alcohol	38·14	36·2
Finland	°alcohol	5·52	39·0	finished product	35·25	13·3	pure alcohol	58·51	39·4
France	°alcohol	4·39	11·9	finished product	10·54	0·5	pure alcohol	34·84	28·0
Georgia	finished product	12·23	5·8	no excise duty	64·22	0·0	finished product	65·83	18·0
Germany	°plato	3·30	3·9	no excise duty	9·51	0·0	pure alcohol	22·57	31·3
Greece	°plato	8·56	12·6	no excise duty	9·96	0·0	pure alcohol	35·97	48·9
Hungary	°alcohol	3·74	15·4	no excise duty	5·92	0·0	pure alcohol	30·63	30·9
Iceland	pure alcohol	11·48	27·7	pure alcohol	49·86	14·3	pure alcohol	72·59	41·0
Ireland	°alcohol	6·51	21·8	finished product	36·26	14·7	pure alcohol	70·35	30·4
Israel	finished product	6·41	9·9	no excise duty	29·06	0·0	pure alcohol	50·92	18·2
Italy	°plato	5·01	10·7	no excise duty	16·13	0·0	pure alcohol	25·31	24·4
Kazakhstan	finished product	3·33	12·5	finished product	19·00	1·3	°alcohol	21·38	35·0
Kosovo	pure alcohol	6·20	19·4	pure alcohol	16·37	11·5	pure alcohol	37·61	9·6
Kyrgyzstan	finished product	7·29	24·7	finished product	18·90	31·8	finished product	24·06	74·8
Latvia	°alcohol	2·87	26·1	finished product	17·34	11·8	pure alcohol	32·30	39·1
Lithuania	°alcohol	3·91	20·0	finished product	19·11	18·9	pure alcohol	35·20	45·8
Luxembourg	°plato	5·59	2·0	no excise duty	21·12	0·0	pure alcohol	29·91	16·5
Malta	°plato	8·36	4·8	finished product	33·86	1·1	pure alcohol	43·88	21·5
Montenegro	°alcohol	8·60	8·5	no excise duty	40·42	0·0	pure alcohol	48·81	35·8
Netherlands	finished product	6·36	7·6	finished product	16·61	6·8	pure alcohol	33·17	25·9
North Macedonia	finished product	4·20	5·1	no excise duty	6·18	0·0	pure alcohol	45·58	15·9
Norway	finished product	6·13	37·5	°alcohol	17·08	37·7	°alcohol	57·23	55·2
Poland	°plato	3·27	18·0	finished product	19·57	5·1	pure alcohol	28·06	51·1
Portugal	finished product	3·37	10·9	no excise duty	10·16	0·0	pure alcohol	26·94	36·3
Republic of Moldova	finished product	4·56	9·9	no excise duty	18·11	0·0	pure alcohol	18·15	39·6
Romania	°plato	3·67	6·8	no excise duty	7·05	0·0	pure alcohol	24·03	34·8
Russia	finished product	4·44	19·3	finished product	13·47	0·1	pure alcohol	23·95	35·3
Serbia	finished product	5·93	10·7	no excise duty	17·68	0·0	finished product	30·35	10·8
Slovakia	°alcohol	3·13	11·4	no excise duty	5·74	0·0	pure alcohol	18·94	45·1
Slovenia	°alcohol	8·06	13·3	no excise duty	18·57	0·0	pure alcohol	32·66	28·6
Spain	finished product	5·65	2·8	no excise duty	6·38	0·0	pure alcohol	25·74	23·8
Sweden	°alcohol	6·87	16·8	finished product	28·66	10·4	pure alcohol	79·57	29·7
Switzerland	finished product	6·66	3·3	no excise duty	16·02	0·0	pure alcohol	42·42	23·8
Tajikistan	finished product	0·54	27·3	finished product	2·62	8·1	pure alcohol	2·90	20·5
Turkey	ad valorem tax	19·71	38·7	finished product	97·46	5·6	pure alcohol	129·31	46·9
Turkmenistan	ad valorem tax	10·75	19·4	ad valorem tax	22·26	23·7	ad valorem tax	37·42	37·9
Ukraine	finished product	4·08	9·6	finished product	12·09	9·4	pure alcohol	25·33	28·4
United Kingdom	°alcohol	6·45	21·7	finished product	20·06	21·8	pure alcohol	52·66	32·1
Uzbekistan	finished product	4·57	1·2	finished product	13·12	0·3	finished product	17·09	3·3

Note: Int$ = International Dollar. Specific tax: excise duty per °alcohol, per °plato, and volume pure alcohol; unitary tax: excise duty per volume of finished product; ad valorem tax: excise duty is levied on basis of final price; no excise duty: no duty exists or excise duty is 0 Int$. Pure alcohol content by beverage: 5% for beer, 12·5% for wine, and 40% for spirits. Missing information indicated by [.].

### 2.2 Taxation scenarios

Similar to Rovira et al. [19], we considered three scenarios in order to estimate the potential effects of a rising taxation: excise duties increased by 20%, 50%, and 100%. For countries that do not levy excise duties on wine (Table 1), the same share of tax rate in mean price as for beer was assumed. Our modelling approach assumes that these increases in the taxation will directly increase the consumer price, and this increase in the price will result in a consumption decrease. The relation between increasing the price and decreasing consumption is given by the price elasticity (Formula 1). Where E is the price elasticity, ∆Q the difference in the percentage of consumption, and ∆P the difference in the percentage of price.

(Formula 1) $$E=\;\frac{\Delta Q}{\Delta P}$$

The value for the elasticity will not be always the same. Previous studies show that despite elasticity values not changing a lot across different countries (e.g., Fogarty explicitly tested stability of elasticities across countries, and concluded, that elasticities are similar across countries [25]), they were found to change considerably across different beverage types [25], [26], [27], dependent on the status of the beverage as being the beverage of choice [28]. We have used, for all countries, an elasticity value equal to -0·36 (95% CI: -0·48, -0·24) for the country's preferred beverage, -1·20 (95% CI: -1·44, -0·96) for the least preferred beverage and -0·60 (95% CI: -0·72, -0·48) for the one in between [28]. We have only considered three beverage types: beer, wine and spirits. These values have been applied to all cases except for heavy drinkers (men drinking >60 g pure alcohol per day and women >40 g pure alcohol per day), where we have applied the value -0·28 (95% CI: -0·37, -0·19) for all beverage types [26]. It is important to make this distinction because heavy drinkers are likely to have a bigger dependence on purchasing alcoholic beverages, which means that the value for the elasticity needs to be closer to 0. Also, as a consequence of this bigger dependence, there will be less differences in elasticity across the different beverage types.

### 2.3 Deriving alcohol-attributable fractions and applying them to cancer incidence and mortality

Model estimates were applied to alcohol exposure data for recorded *per capita* consumption for the population aged 15 years and older in 2009, derived from a global modelling study [29], assuming a lag time of ten years between alcohol intake and cancer development and mortality [30]. Based on the recommendations of the WHO Technical Advisory Group on Alcohol and Drug Epidemiology, we have only used 80% of the 2009 *per capita* consumption values. The 80% is based on two reasons: first, as not all alcohol is consumed, but some is spilled and left in the bottle, and second, to be conservative in adjusting for the underreporting of alcohol in medical epidemiological studies [31]. Based on the estimated reduced alcohol consumption, we determined alcohol-attributable fractions for each cancer type and compared them to the alcohol-attributable fractions in the baseline scenario (i.e., current excise duties). Comparisons between modelled alcohol-attributable fractions with those from the baseline scenario were categorised by sex and age for the different scenarios and for all cancers causally linked to alcohol. The latter were based on the classification of the International Agency for Research on Cancer, taking only cancer types with sufficient evidence of a causal relationship with alcohol consumption: [4,8] lip and oral cavity cancer (Global Burden of Disease Study [GBD] codes: C00-C07, C08-C08.9, Z85.81-Z85.810), pharynx cancer (GBD codes: C09-C10.9, C12-C13.9), oesophagus cancer (GBD codes: C15-C15.9, Z85.01), colon and rectum cancers (GBD codes: C18-C19.0, C20, C21-C21.8, Z12.1-Z12.13, Z85.03-Z85.048, Z86.010), liver cancer (GBD codes: C22-C22.4, C22.7-C22.9, Z85.05), larynx cancer (GBD codes: C32-C32.9, Z85.21), and female breast cancer (GBD codes: C50-C50.629, C50.8-C50.929, Z12.3-Z12.39, Z80.3, Z85.3, Z86.000). In case of oesophageal cancer, a causal relationship has been established for squamous cell carcinomas only, so country-specific data on the proportion of squamous cell carcinomas in all oesophageal cancers were obtained from the International Agency for Research on Cancer and applied to the GBD estimates of oesophageal cancer incidence and mortality (same as in [7]). We further calculated the proportion of potentially avoidable new cancer cases and deaths from the different tax increase scenarios out of all alcohol-attributable cancers (those estimated to have been caused by alcohol) and all alcohol-related cancers (all new cases or deaths for cancer types whose risk is increased by alcohol consumption). A Monte Carlo simulation with 1,000 repetitions was used to estimate the 95% confidence intervals [32].

Data on incident cancer cases and deaths for 2019 were obtained from the Global Health Data Exchange website, based on the Global Burden of Disease 2019 Study [33]. Relevant risk functions required for the calculation of alcohol-attributable fractions were taken from Shield et al [34].

### 2.4 Sensitivity analysis

We conducted two different types of sensitivity analyses. A first using an alternative tax increase scenario in order to evaluate the effect of all countries applying the same tax rates as Finland, and a second one taking into account a lag time of 20 years. Modelling an alternative tax increase scenario, Finland was chosen as the country with the highest share of tax rate in mean price on beer. With regard to the second sensitivity analysis, we repeated modelling assuming a lag time of 20 years between alcohol exposure and cancer development and mortality, which is the upper limit for lag time based on a systematic review [35]. We therefore used recorded *per capita* consumption data from 1999 for all countries, except Montenegro and Serbia. For these two countries, which only became independent in 2006, consumption data was not available for 1999, so we applied the latest available data from 2006.

### 2.5 Role of the funding source

This project was designed and implemented without external financial funding.

## 3 Results

Within the entire WHO European Region, there have been almost 1·4 million (95% confidence intervals [CI]: 1,376,865-1,414,327) new alcohol-related cancer cases and 649,814 (95% CI: 642,861-655,793) alcohol-related deaths in 2019. Of those, 180,887 (95% CI: 160,595-201,705) cancer cases and 85,130 (95% CI: 74,920-95,523) deaths were estimated to be caused by alcohol.

Increasing excise duties on alcoholic beverages by 20%, 50%, and 100% could have potentially been avoided 1·2% (95% CI: 1·1-1·2), 2·9% (95% CI: 2·8-3·1), and 5·9% (95% CI: 5·6-6·4) of new alcohol-attributable cancer cases, respectively, or up to 10,716 (95% CI: 9,433-12,339) new cancer cases (see Table 2). With regards to mortality, 5·7% (95% CI: 5·4-6·1) of alcohol-attributable cancer deaths or 4,846 (95% CI: 4,219-5,563) deaths could have been averted by increasing excise duties by 100%. Relative to alcohol-related cancers estimated in the Region in 2019, up to 0·8% (95% CI: 0·7-0·9) of new cancer cases and 0·7% (95% CI: 0·6-0·9) of cancer deaths could have been avoided in the 100% tax increase scenario.

**Table 2:** Avoidable new alcohol-attributable cancer cases and deaths in 2019 for the different tax increase scenarios for the entire WHO European Region.

Taxation increase scenario†	Cancer incidence			Cancer deaths		
	Total number of avoided cancers	% alcohol-attributable cancers‡	% alcohol-related cancers§	Total number of avoided cancers	% alcohol-attributable cancers‡	% alcohol-related cancers§
20%	2,096 (1,847-2,390)	1·2 (1·1-1·2)	0·2 (0·1-0·2)	944 (821-1,082)	1·1 (1·0-1·2)	0·1 (0·1-0·2)
50%	5,283 (4,654-6,028)	2·9 (2·8-3·1)	0·4 (0·3-0·4)	2,383 (2,073-2,733)	2·8 (2·6-3·0)	0·4 (0·3-0·4)
100%	10,716 (9,433-12,239)	5·9 (5·6-6·4)	0·8 (0·7-0·9)	4,846 (4,219-5,563)	5·7 (5·4-6·1)	0·7 (0·6-0·9)

† Tax increase on national beverage-specific alcohol excise duties on beer, wine, and spirits.

‡ Alcohol-attributable cancers refer to those new cancer cases or deaths estimated to have been caused by alcohol.

§ Alcohol-related cancers refer to all new cases or deaths for cancers whose risk is increased by alcohol consumption.

**Fig. 1:**
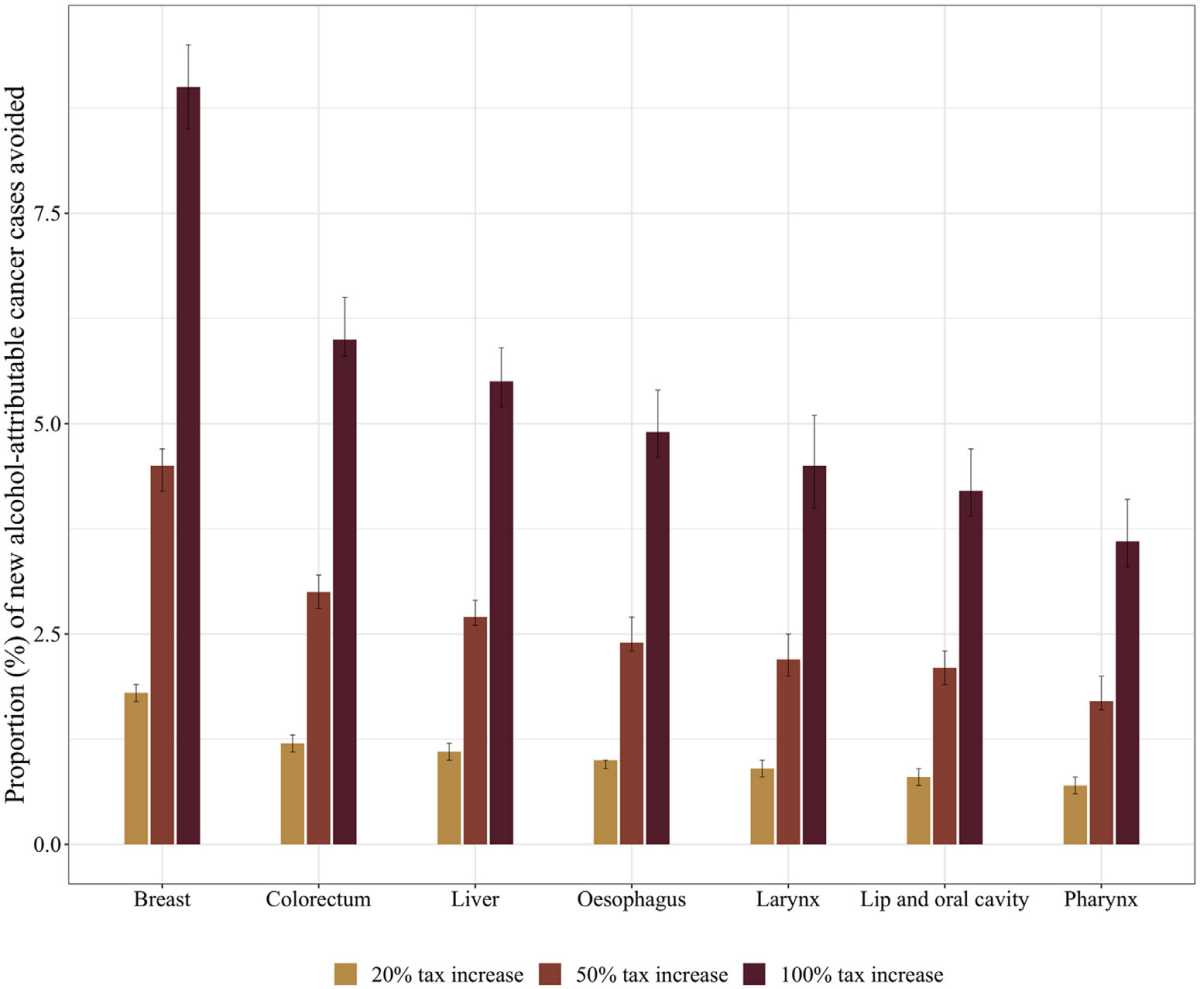
Proportion and 95% confidence intervals of new alcohol-attributable cancer cases that would have been avoided by cancer site and taxation increase scenario in 2019 for the entire WHO European Region. In oesophageal cancers, only cases of squamous cell carcinoma were considered.

Comparing different cancer sites (Fig. 1), the highest number of new cancer cases and deaths could have been averted for breast and colorectal cancers, both being the most prevalent alcohol-related cancer sites in the Region [10]. For the 100% tax increase scenario, this equated to 1,086 (95% CI: 868-1,318) lives potentially saved in women and 1,770 (95% CI: 1,260-2,310) lives potentially saved in women and men for breast and colorectal cancer, respectively. For the remaining cancer sites, roughly 3·5% to 5·5% of alcohol-attributable new cancer cases and deaths could have potentially been avoided if taxes were increased by 100% (see Supplementary Table S3).

**Fig. 2:**
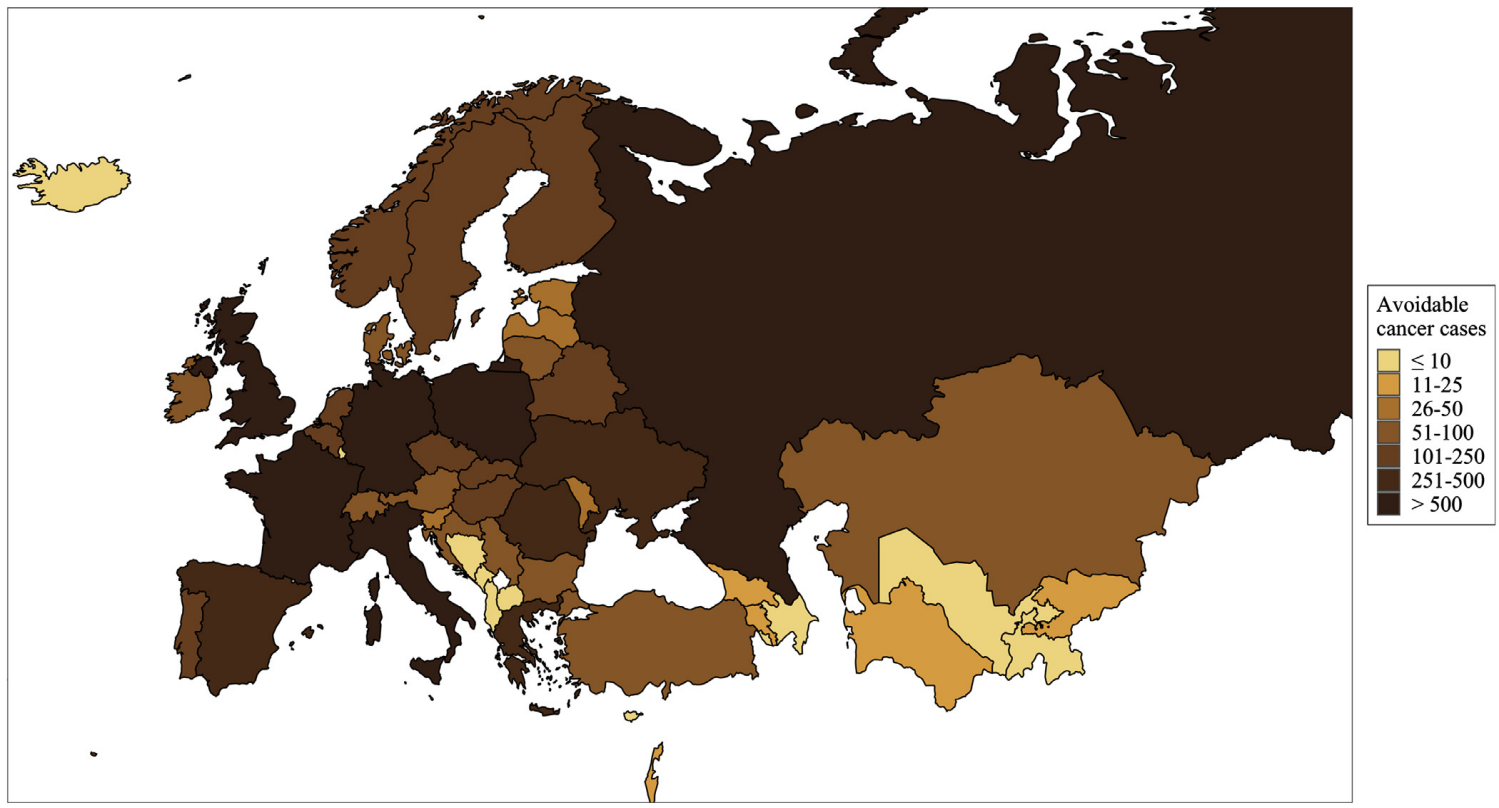
Total number of new cancer cases that would have been avoided if national alcohol excise duties were increased by 100%.

**Fig. 3:**
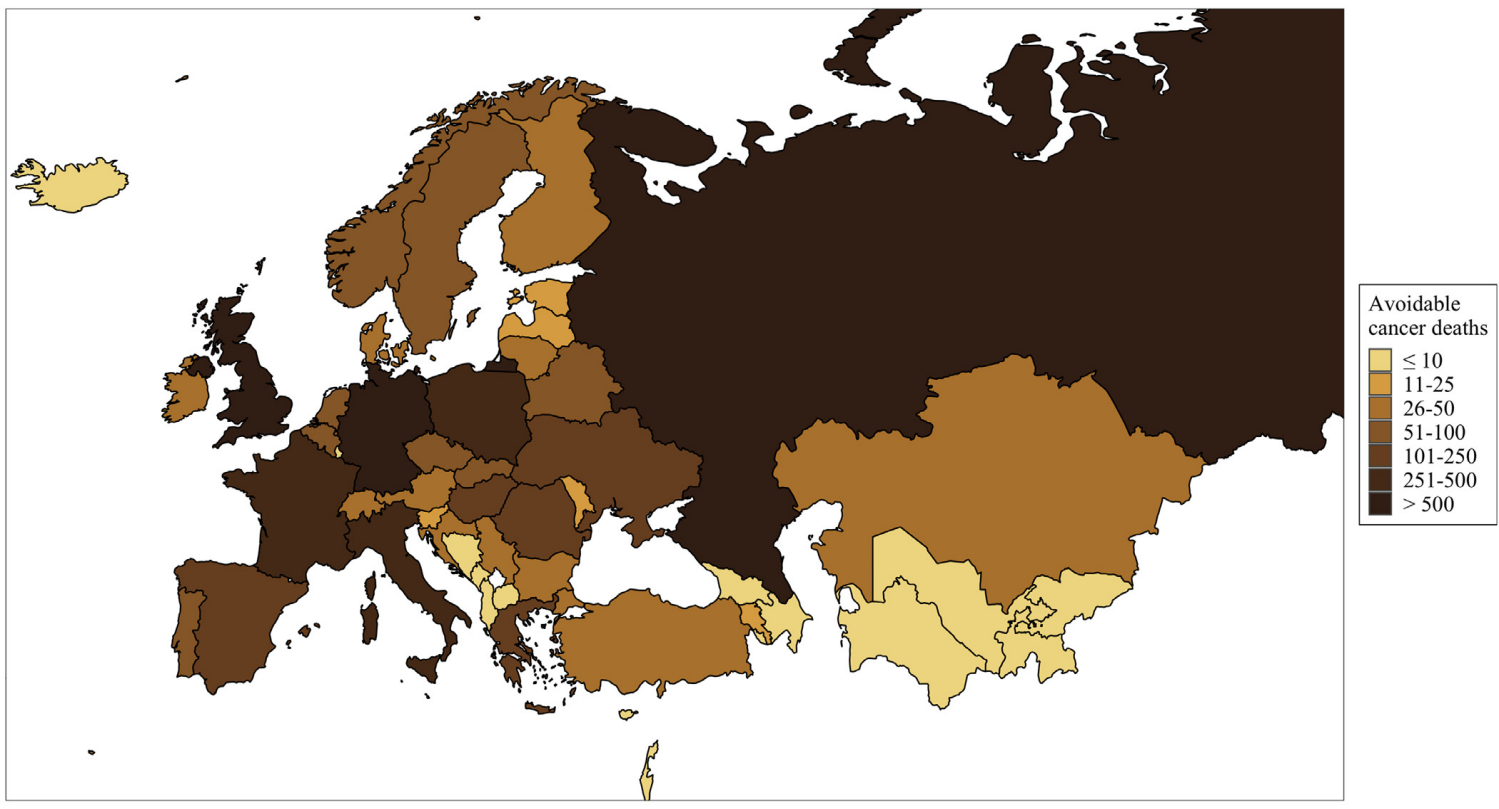
Total number of cancer deaths that would have been avoided if national alcohol excise duties were increased by 100%.

The regional distribution of estimated avoidable new cancer cases and deaths for the 100% tax increase scenario is shown in Fig. 2, Fig. 3, respectively (for CIs and other tax increase scenarios, see Supplementary Table S4). The highest total numbers of potentially avoidable new alcohol-attributable cancer cases and deaths were estimated in the UK (1,813, 95% CI: 1,496-2,262, and 681, 95% CI: 558-864, respectively), Russia (1,414, 95% CI: 1,090-1,819, and 727, 95% CI: 561-938, respectively), and Germany (1,268, 95% CI: 1,008-1,560, and 529, 95% CI: 421-656, respectively). However, the highest proportion of such new cancer cases and deaths could have been avoided in Norway (23·7%, 95% CI: 19·8-27·6 and 23·8%, 95% CI: 19·7-27·9, respectively), Armenia (16·9%, 95% CI: 11·9-21·5 and 16·8%, 95% CI: 11·8-21·4, respectively), and Iceland (14·3%, 95% CI: 12·2-16·5 and 14·2%, 95% CI: 12·1-16·4, respectively). In contrast, this proportion was lowest in Bosnia and Herzegovina, Luxembourg, and Uzbekistan, where less than 3% of new alcohol-attributable cancers and deaths could have potentially been avoided.

When applying Finland's current excise duties to all WHO European countries modelled, 9,123 (95% CI: 7,975-10,389) new cancer cases and 4,051 (95% CI: 3,525-4,633) deaths could have potentially been avoided. This equates to 5·0% (95% CI: 4·8-5·4) and 4·8% (95% CI: 4·5-5·1) of new alcohol-attributable cancer cases and deaths in the Region, respectively. Estimates by cancer type and by WHO European Region Member State are presented in the Supplementary Tables S5 and S6, respectively. In countries that levy higher alcohol excise duties on beer, wine, or spirits than in Finland, the number of avoidable cancers can be lower than zero if the relevant alcoholic beverage account for a relatively high proportion of *per capita* consumption (i.e., Armenia, Kyrgyzstan, Norway, and Turkey).

Assuming a lag time of 20 years between alcohol exposure and cancer development and deaths resulted in slightly lower numbers of avoidable new cancer cases and deaths (see Supplementary Table S7). The proportional change in the number of avoidable new cancer cases assuming a 10- versus 20-year lag period varied between 2·0% in lip and oral cavity cancer and 4·8% in liver cancer, and between less than 0·1% in pharynx cancer and 6·4% in breast cancer for alcohol-attributable cancer deaths.

## 4 Discussion

Increasing excise duties on alcoholic beverages in the Member States of the WHO European Region could potentially avert new alcohol-attributable cancer cases and deaths. Our modelling study revealed that almost 11,000 cases or 5·9% of new alcohol-attributable cancer cases and about 5,000 deaths or 5·7% of alcohol-attributable cancer deaths could have been avoided in one year, 2019, if national excise duties were increased by 100%. Relative to the total population of the Region [36], this translates into 11·6 avoidable new cancer cases per million population and 5·2 lives saved per million population in one year. For smaller tax increases, a relatively lower number of new cancer cases and deaths caused by alcohol could have been avoided.

Before discussing our results, we will address the potential limitations imposed by the assumptions underlying the model. While our modelling approach considered the sex- and age-specific impact of alcohol use on cancer, the distributions of beverage types were assumed to be the same across groups, thus assuming no shifts based on sex-specific elasticities and cross-elasticities of alcohol [25,37]. Though a recent modelling study has demonstrated that accounting for sex-specific price elasticity has only a limited impact on the modelled effects of pricing policies on alcohol consumption and harm [37], meta-analytical investigation of sex-specific price elasticities is as yet lacking. Additional bias may result from not modelling the cross-elasticities between alcoholic beverages or between alcohol and other substances [38], and the assumption that the increased price would be fully transferred to the consumer, which is supported by findings from meta-analysis [39]. Furthermore, we have assumed the relative risk function to be the same in all countries, which may be a simplification as drinking patterns vary [40]. The dose-response curve between alcohol and cancer, however, has been shown to be stable with respect to the impact of drinking patterns [41]. *Per capita* consumption data are unlikely to introduce much bias as only recorded consumption data that has a high degree of validity have been considered. However, unrecorded consumption is estimated to account for a considerable share of total *per capita* consumption in some countries such as Greece, Kyrgyzstan, and Uzbekistan [14], and whether increases in excise duties could lead to an increase in unrecorded consumption is a matter of concern that needs to be considered in policy implementation [42,43]. However, a recent review showed, that, empirically, there did not seem to be a systematic increase in unrecorded consumption following taxation increases [44]. Additionally, the consideration of sex- and age-specific *per capita* consumption data may lead to some bias in the exposure data, as these rely on surveys and thus may be subject to underreporting [45]. Finally, we used current data on prices and excise duties for alcoholic beverages as baseline scenario and applied the model estimates to 2009 exposure data in order to account for a 10-years lag time, and in sensitivity analysis to 1999 exposure data accounting for a 20-years lag time, between alcohol exposure and cancer development and mortality. Lag times were selected based on those conventionally used in modelling cancer incidences [7,34], however, they cannot be applied to the individual case as the time between exposure and cancer development and mortality varies from individual to individual. Eventually, both models provide conservative estimates in the sense that, assuming the taxation increase continue to impact consumption after one year, more new cancer cases and deaths would be prevented. Given the long average lag time between alcohol exposure and different cancer outcomes [30], we would like to emphasise that no immediate change in cancer burden would be expected if current excise duties were increased.

A substantial number of new cancer cases and deaths in seven different cancer sites causally linked to alcohol have been estimated to be avoidable by increasing current national excise duties, with breast and colorectal cancers being the most common alcohol-related cancers in the Region [10]. Breast cancer takes on a particularly important role, as the risk is sharply increased even with small daily amounts of pure alcohol. About half of alcohol-attributable breast cancer cases in the EU are caused by light to moderate alcohol consumption [13], which underlines the need for measures to reduce alcohol consumption of any level of intake among the population. Additionally, public awareness of the cancer risk posed by alcohol is generally low [46,47], and misconceptions exist, for example, that only some alcoholic beverages or heavy drinking would lead to the development of cancers [48,49]. We believe that our findings are important in informing the public as well as policy makers about the cancer risk posed by alcohol, empowering them to make informed decisions about their individual consumption and alcohol policies, respectively. If current excise duties were increased by 100%, almost one in ten new alcohol-attributable breast cancer cases could have potentially been avoided. As breast cancer, alongside colorectal cancer, are the most commonly diagnosed cancers in the WHO European Region, together representing more than one million cases in 2020 [6], any gains to be made in lessening the burden of these cancers through policy changes must be explored further.

The impact of the different tax increase scenarios varies across countries and depends on current excise duties and the incidence of alcohol-related cancers. The number of potentially avoidable cancer cases and deaths is particularly high in countries such as France, Germany, Italy, and Spain, where cancer prevalence and alcohol *per capita* consumption are relatively high and current excise duties are low or even zero, as for wine [24]. While a 100% increase in excise duty may appear to be unrealistically high, a doubling of current excise duties in most countries would still keep tax rates, particularly for beer and wine, below those in Finland, which was selected as good practice example in the sensitivity analysis. The case of Finland demonstrates that a reasonably high level of taxation is possible, and our sensitivity analysis exemplifies the potential impact of such a tax increase on the cancer burden in the Region. Additional support in favour of achieving substantial tax increases comes from Lithuania, where excise duties on beer and wine were doubled in 2017 [50]. With Europe's Beating Cancer Plan, the EU has committed to review the current alcohol tax legislation to support cancer control programmes in their Member States [51]. Therefore, there is a window of opportunity for a reduction in the alcohol-related cancer burden by increasing excise duties on alcoholic beverages.

In addition to increasing alcohol excise duties, as modelled in our study, other considerations for successful tax implementation need to be taken into account to effectively reduce the alcohol-attributable cancer burden [15,17]. First, the taxation of both beer and wine is in many countries independent of the alcohol content but based on the volume of the finished product (i.e., unitary tax, see Table 1) and therefore does not reflect the amount of its carcinogenic component ethanol (like it is in the case of a specific or volumetric tax). Volumetric taxation would not only set excise duties at a scientifically substantiated level, but might also have a greater impact on heavy drinking individuals, who tend to consume stronger and cheaper alcohol [52]. Second, excise duties are often not linked to inflation, as a result of which their effect on alcohol affordability decreases with increasing inflation, meaning that alcohol is becoming more affordable in many countries [17]. Differences between alcohol taxation policies in countries will also show how policymakers respond to the challenge of meeting health care needs, notably the costs imposed by increased cancer rates, knowing that a high number of cancer cases and deaths could be prevented. Next to reducing alcohol-attributable cancers, an increase in alcohol excise duties would also impact other alcohol-attributable diseases and injuries [53], bringing benefits to the population at large, including young people, where alcohol-attributable burden of disease is proportionally highest [54]. Finally, alcohol taxation appears to be a promising policy option to reduce the cancer burden caused by alcohol, and should sought as part of a comprehensive alcohol policy strategy in order to tackle the alcohol-attributable burden of disease in the Region, including and beyond cancer.

## Data sharing statement

All relevant data were obtained from publicly available sources as referenced in the methods section or published with this report. The statistical codes supporting the results of this study are available from the corresponding author [C.K.]

## Editor note

The Lancet Group takes a neutral position with respect to territorial claims in published maps and institutional affiliations.

## Declaration of interests

The authors have no conflicts of interest to declare. Carina Ferreira-Borges is a staff member of WHO, Maria Neufeld is a WHO consultant. Where authors are identified as personnel of the International Agency for Research on Cancer and WHO, the authors alone are responsible for the views expressed in this article and they do not necessarily represent the decisions, policy, or views of the International Agency for Research on Cancer and WHO.

## Appendix Supplementary materials

Image, application 1

Image, application 2

## Footnotes

Supplementary material associated with this article can be found in the online version at doi:10.1016/j.lanepe.2021.100225.
