# Supplementary material for: Modelling the impact of increased alcohol taxation on alcohol-attributable cancers in the WHO European Region
Source: Lancet Reg Health Eur. 2021 Sep 15;11:100225. doi: 10.1016/j.lanepe.2021.100225 (PMC8642705; doi:10.1016/j.lanepe.2021.100225)
Supplement: Supplementary file 1 [file mmc1.docx]

**Supplementary material**

**Title:** Modelling the impact of increased alcohol taxation on alcohol-attributable cancers in the WHO European Region

**Authors:** Carolin Kilian, Pol Rovira, Maria Neufeld, Carina Ferreira-Borges, Harriet Rumgay, Isabelle Soerjomataram, Jürgen Rehm

[Supplementary Table S1. Checklist of information (GATHER) that should be included in new reports of global health estimates 2](#_Toc79597682)

[Supplementary Table S2. Source excise duties and mean prices by country; data obtained Nov 2020 3](#_Toc79597683)

[Supplementary Table S3. Avoidable new alcohol-attributable cancer cases and deaths by cancer site for different tax-increase scenarios for the entire WHO European Region. 5](#_Toc79597684)

[Supplementary Table S4. Avoidable new alcohol-attributable cancer cases and deaths for different tax-increase scenarios by countries of the WHO European Region. 6](#_Toc79597685)

[Supplementary Table S5. Sensitivity analysis 1: Avoidable new alcohol-attributable cancer cases and deaths for applying the same tax rate as Finland, entire WHO European Region. 11](#_Toc79597686)

[Supplementary Table S6. Sensitivity analysis 1: Avoidable new alcohol-attributable cancer cases and deaths for applying the same tax rate as Finland, by countries of the WHO European Region. 12](#_Toc79597687)

[Supplementary Table S7. Sensitivity analysis 2: Avoidable new alcohol-attributable cancer cases and deaths for each tax increase scenario by cancer site and for the entire WHO European Region, applying a lag time between alcohol exposure and cancer development or deaths of 20 years. 14](#_Toc79597688)

Supplementary Table S1. Checklist of information (GATHER) that should be included in new reports of global health estimates

| Item # | Checklist item | Reported on page # |
| --- | --- | --- |
| Objectives and funding | | |
| 1 | Define the indicator(s), populations (including age, sex, and geographic entities), and time period(s) for which estimates were made. | M5-M6 |
| 2 | List the funding sources for the work. | M7 |
| Data Inputs | | |
| *For all data inputs from multiple sources that are synthesized as part of the study:* | | |
| 3 | Describe how the data were identified and how the data were accessed. | M4-M7 |
| 4 | Specify the inclusion and exclusion criteria. Identify all ad-hoc exclusions. | M5 |
| 5 | Provide information on all included data sources and their main characteristics. For each data source used, report reference information or contact name/institution, population represented, data collection method, year(s) of data collection, sex and age range, diagnostic criteria or measurement method, and sample size, as relevant. | M4-M7 |
| 6 | Identify and describe any categories of input data that have potentially important biases (e.g., based on characteristics listed in item 5). | M12-M13 |
| *For data inputs that contribute to the analysis but were not synthesized as part of the study:* | | |
| 7 | Describe and give sources for any other data inputs. | Table S2 |
| *For all data inputs:* | | |
| 8 | Provide all data inputs in a file format from which data can be efficiently extracted (e.g., a spreadsheet rather than a PDF), including all relevant meta-data listed in item 5. For any data inputs that cannot be shared because of ethical or legal reasons, such as third-party ownership, provide a contact name or the name of the institution that retains the right to the data. | Table 1 (M) |
| Data analysis | | |
| 9 | Provide a conceptual overview of the data analysis method. A diagram may be helpful. | M5-M7 |
| 10 | Provide a detailed description of all steps of the analysis, including mathematical formulae. This description should cover, as relevant, data cleaning, data pre-processing, data adjustments and weighting of data sources, and mathematical or statistical model(s). | M5-M7 |
| 11 | Describe how candidate models were evaluated and how the final model(s) were selected. | M6-M7 |
| 12 | Provide the results of an evaluation of model performance, if done, as well as the results of any relevant sensitivity analysis. | M7, M11-M12 |
| 13 | Describe methods for calculating uncertainty of the estimates. State which sources of uncertainty were, and were not, accounted for in the uncertainty analysis. | M7 |
| 14 | State how analytic or statistical source code used to generate estimates can be accessed. | M15 |
| Results and Discussion | | |
| 15 | Provide published estimates in a file format from which data can be efficiently extracted. | Table 2 (M), Tables S3-S7 |
| 16 | Report a quantitative measure of the uncertainty of the estimates (e.g. uncertainty intervals). | Table 2 (M), Tables S3-S7 |
| 17 | Interpret results in light of existing evidence. If updating a previous set of estimates, describe the reasons for changes in estimates. | M12-15 |
| 18 | Discuss limitations of the estimates. Include a discussion of any modelling assumptions or data limitations that affect interpretation of the estimates. | M12-M13 |
| Note. M = Manuscript, S = Supplementary material. | | |

Supplementary Table S2. Source excise duties and mean prices by country; data obtained Nov 2020

| **Country** | **Source: Excise duties** | **Source: Mean prices alcoholic beverages** |
| --- | --- | --- |
| Albania | <http://www.dogana.gov.al/english/dokument/462/ndryshim-6-ligj-126-2016-1512201> | <https://www.statista.com/outlook/cmo/alcoholic-drinks/worldwide> |
| Armenia | <https://www.arlis.am/DocumentView.aspx?docid=137404> | <https://www.armstat.am/am/?nid=82> |
| Austria | <https://ec.europa.eu/taxation_customs/business/excise-duties-alcohol-tobacco-energy/excise-duties-alcohol_en> | <https://www.statista.com/outlook/cmo/alcoholic-drinks/worldwide> |
| Azerbaijan | <http://continent-online.com/Document/?doc_id=30414629#pos=2866;-52&sdoc_params=text%3D%25D0%25B2%25D0%25BE%25D0%25B4%25D0%25BA%25D0%25B0%26mode%3Dindoc%26topic_id%3D30414629%26spos%3D1%26tSynonym%3D1%26tShort%3D1%26tSuffix%3D1&sdoc_pos=0> | <http://www.stat.gov.mk/Publikacii/SG2020/SG2020-Pdf/08-PrihodiPotrosCeni-IncomeExpPrices.pdf> |
| Belarus | <http://www.minfin.gov.by/ru/news/ba9bc2523a5047bd.html> | <https://www.belstat.gov.by/ofitsialnaya-statistika/realny-sector-ekonomiki/tseny/potrebitelskie-tseny/operativnye-dannye/srednie-tseny-na-potrebitelskie-tovary-i-uslugi-po-respublike-belarus/> |
| Belgium | <https://ec.europa.eu/taxation_customs/business/excise-duties-alcohol-tobacco-energy/excise-duties-alcohol_en> | <https://www.statista.com/outlook/cmo/alcoholic-drinks/worldwide> |
| Bosnia and Herzegovina | <https://advokat-prnjavorac.com/legislation/Law-on-excise-duties-FBiH.pdf> | <https://www.statista.com/outlook/cmo/alcoholic-drinks/worldwide> |
| Bulgaria | <https://ec.europa.eu/taxation_customs/business/excise-duties-alcohol-tobacco-energy/excise-duties-alcohol_en> | <https://www.statista.com/outlook/cmo/alcoholic-drinks/worldwide> |
| Croatia | <https://ec.europa.eu/taxation_customs/business/excise-duties-alcohol-tobacco-energy/excise-duties-alcohol_en> | <https://www.statista.com/outlook/cmo/alcoholic-drinks/worldwide> |
| Cyprus | <https://ec.europa.eu/taxation_customs/business/excise-duties-alcohol-tobacco-energy/excise-duties-alcohol_en> | <https://www.statista.com/outlook/cmo/alcoholic-drinks/worldwide> |
| Czechia | <https://ec.europa.eu/taxation_customs/business/excise-duties-alcohol-tobacco-energy/excise-duties-alcohol_en> | <https://www.statista.com/outlook/cmo/alcoholic-drinks/worldwide> |
| Denmark | <https://ec.europa.eu/taxation_customs/business/excise-duties-alcohol-tobacco-energy/excise-duties-alcohol_en> | <https://www.statista.com/outlook/cmo/alcoholic-drinks/worldwide> |
| Estonia | <https://ec.europa.eu/taxation_customs/business/excise-duties-alcohol-tobacco-energy/excise-duties-alcohol_en> | <https://www.statista.com/outlook/cmo/alcoholic-drinks/worldwide> |
| Finland | <https://ec.europa.eu/taxation_customs/business/excise-duties-alcohol-tobacco-energy/excise-duties-alcohol_en> | <https://www.statista.com/outlook/cmo/alcoholic-drinks/worldwide> |
| France | <https://ec.europa.eu/taxation_customs/business/excise-duties-alcohol-tobacco-energy/excise-duties-alcohol_en> | <https://www.statista.com/outlook/cmo/alcoholic-drinks/worldwide> |
| Georgia | <https://www.matsne.gov.ge/ka/document/view/1043717?impose=translateEn&publication=118> | <https://www.statista.com/outlook/cmo/alcoholic-drinks/worldwide> |
| Germany | <https://ec.europa.eu/taxation_customs/business/excise-duties-alcohol-tobacco-energy/excise-duties-alcohol_en> | <https://www.statista.com/outlook/cmo/alcoholic-drinks/worldwide> |
| Greece | <https://ec.europa.eu/taxation_customs/business/excise-duties-alcohol-tobacco-energy/excise-duties-alcohol_en> | <https://www.statista.com/outlook/cmo/alcoholic-drinks/worldwide> |
| Hungary | <https://ec.europa.eu/taxation_customs/business/excise-duties-alcohol-tobacco-energy/excise-duties-alcohol_en> | <https://www.statista.com/outlook/cmo/alcoholic-drinks/worldwide> |
| Iceland | <https://ec.europa.eu/neighbourhood-enlargement/sites/near/files/pdf/iceland/key-documents/screening_report_16_is_internet_en.pdf> | <https://www.statista.com/outlook/cmo/alcoholic-drinks/worldwide> |
| Ireland | <https://ec.europa.eu/taxation_customs/business/excise-duties-alcohol-tobacco-energy/excise-duties-alcohol_en> | <https://www.statista.com/outlook/cmo/alcoholic-drinks/worldwide> |
| Israel | <https://www.keepeek.com//Digital-Asset-Management/oecd/taxation/consumption-tax-trends-2020_152def2d-en#page156> | <https://www.statista.com/outlook/cmo/alcoholic-drinks/worldwide> |
| Italy | <https://ec.europa.eu/taxation_customs/business/excise-duties-alcohol-tobacco-energy/excise-duties-alcohol_en> | <https://www.statista.com/outlook/cmo/alcoholic-drinks/worldwide> |
| Kazakhstan | <https://online.zakon.kz/document/?doc_id=36148637#pos=11003;35&sdoc_params=text%3D%25D0%25A1%25D0%25BF%25D0%25B8%25D1%2580%25D1%2582%25D0%25BE%25D1%2581%25D0%25BE%25D0%25B4%25D0%25B5%25D1%2580%25D0%25B6%25D0%25B0%25D1%2589%25D0%25B0%25D1%258F%2520%25D0%25BF%25D1%2580%25D0%25BE%25D0%25B4%25D1%2583%25D0%25BA%25D1%2586%25D0%25B8%25D1%258F%2520%25D0%25BC%25D0%25B5%25D0%25B4%25D0%25B8%25D1%2586%25D0%25B8%25D0%25BD%25D1%2581%25D0%25BA%25D0%25BE%25D0%25B3%25D0%25BE%26mode%3Dindoc%26topic_id%3D36148637%26spos%3D1%26tSynonym%3D1%26tShort%3D1%26tSuffix%3D1&sdoc_pos=0> | <https://3pulse.com/en/geo/kazakhstan/prices> |
| Kosovo | <https://mf.rks-gov.net/desk/inc/media/080465ED-A868-4CA0-ADE1-21E8CE9C39FF.pdf>; <https://mf.rks-gov.net/desk/inc/media/D0893084-0E73-476E-8C43-FE8C1E059C3B.pdf?fbclid=IwAR2Pku3MkbfeM0_9a37b-m0vZUHYqynToG-0e-HpFkANhg2wIM4CM4csSIg> | <https://www.belstat.gov.by/ofitsialnaya-statistika/realny-sector-ekonomiki/tseny/potrebitelskie-tseny/operativnye-dannye/srednie-tseny-na-potrebitelskie-tovary-i-uslugi-po-respublike-belarus/> |
| Kyrgyzstan | <https://online.zakon.kz/document/?doc_id=30355506&doc_id2=30355506#activate_doc=2&pos=129;-98&pos2=4115;-94> | <http://www.stat.kg/ru/statistics/ceny-i-tarify/> |
| Latvia | <https://ec.europa.eu/taxation_customs/business/excise-duties-alcohol-tobacco-energy/excise-duties-alcohol_en> | <https://www.statista.com/outlook/cmo/alcoholic-drinks/worldwide> |
| Lithuania | <https://ec.europa.eu/taxation_customs/business/excise-duties-alcohol-tobacco-energy/excise-duties-alcohol_en> | <https://www.statista.com/outlook/cmo/alcoholic-drinks/worldwide> |
| Luxembourg | <https://ec.europa.eu/taxation_customs/business/excise-duties-alcohol-tobacco-energy/excise-duties-alcohol_en> | <https://www.statista.com/outlook/cmo/alcoholic-drinks/worldwide> |
| Malta | <https://ec.europa.eu/taxation_customs/business/excise-duties-alcohol-tobacco-energy/excise-duties-alcohol_en> | <https://www.statista.com/outlook/cmo/alcoholic-drinks/worldwide> |
| Montenegro | <https://mif.gov.me/ResourceManager/FileDownload.aspx?rid=285399&rType=2&file=Law%20on%20Excise%20Taxes%20-%20OGMN%2050-2017%20ENG.DOCX> | <https://www.statista.com/outlook/cmo/alcoholic-drinks/worldwide> |
| Netherlands | <https://ec.europa.eu/taxation_customs/business/excise-duties-alcohol-tobacco-energy/excise-duties-alcohol_en> | <https://www.statista.com/outlook/cmo/alcoholic-drinks/worldwide> |
| North Macedonia | <https://customs.gov.mk/index.php/en/biznis-zaednica-mk-2/akcizi/alkohol-i-alkoholni-pijaloci> | <http://www.stat.gov.mk/Publikacii/SG2020/SG2020-Pdf/08-PrihodiPotrosCeni-IncomeExpPrices.pdf> |
| Norway | <https://www.skatteetaten.no/globalassets/bedrift-og-organisasjon/avgifter/saravgifter/alkoholholdige-drikkevarer/2020-alkohol-ii-juli.pdf>; <https://www.drikkeglede.no/om-oss/brod-i-tall/avgifter/> | <https://www.vinmonopolet.no/> |
| Poland | <https://ec.europa.eu/taxation_customs/business/excise-duties-alcohol-tobacco-energy/excise-duties-alcohol_en> | <https://www.statista.com/outlook/cmo/alcoholic-drinks/worldwide> |
| Portugal | <https://ec.europa.eu/taxation_customs/business/excise-duties-alcohol-tobacco-energy/excise-duties-alcohol_en> | <https://www.statista.com/outlook/cmo/alcoholic-drinks/worldwide> |
| Republic of Moldova | <http://lex.md/fisc/codfiscaltxtru.htm> | <http://www.stat.kg/ru/statistics/ceny-i-tarify/> |
| Romania | <https://ec.europa.eu/taxation_customs/business/excise-duties-alcohol-tobacco-energy/excise-duties-alcohol_en> | <https://www.statista.com/outlook/cmo/alcoholic-drinks/worldwide> |
| Russian Federation | <http://www.consultant.ru/document/cons_doc_LAW_28165/22201a65e4f59a582714243c15b655989bd57066/> | <https://rosstat.gov.ru/price> |
| Serbia | <https://www.carina.rs/cyr/Zakoni/%D0%A3%D1%81%D0%BA%D0%BB%D0%B0%D1%92%D0%B5%D0%BD%D0%B8%20%D0%B4%D0%B8%D0%BD%D0%B0%D1%80%D1%81%D0%BA%D0%B8%20%D0%B8%D0%B7%D0%BD%D0%BE%D1%81%D0%B8%20%D0%B0%D0%BA%D1%86%D0%B8%D0%B7%D0%B0%20%D0%BE%D0%B4%201.7.2020.pdf> | <https://publikacije.stat.gov.rs/G2020/Pdf/G20202053.pdf> |
| Slovakia | <https://ec.europa.eu/taxation_customs/business/excise-duties-alcohol-tobacco-energy/excise-duties-alcohol_en> | <https://www.statista.com/outlook/cmo/alcoholic-drinks/worldwide> |
| Slovenia | <https://ec.europa.eu/taxation_customs/business/excise-duties-alcohol-tobacco-energy/excise-duties-alcohol_en> | <https://www.statista.com/outlook/cmo/alcoholic-drinks/worldwide> |
| Spain | <https://ec.europa.eu/taxation_customs/business/excise-duties-alcohol-tobacco-energy/excise-duties-alcohol_en> | <https://www.statista.com/outlook/cmo/alcoholic-drinks/worldwide> |
| Sweden | <https://ec.europa.eu/taxation_customs/business/excise-duties-alcohol-tobacco-energy/excise-duties-alcohol_en> | <https://www.statista.com/outlook/cmo/alcoholic-drinks/worldwide> |
| Switzerland | <https://www.ezv.admin.ch/ezv/de/home/themen/alcohol/steuersaetze.html> | <https://www.statista.com/outlook/cmo/alcoholic-drinks/worldwide> |
| Tajikistan | <https://online.zakon.kz/Document/?doc_id=34791241#pos=1;-80> [Excise tax is fixed in EUR currency in legislation] | <https://www.statista.com/outlook/cmo/alcoholic-drinks/worldwide> |
| Turkey | <https://www.gib.gov.tr/fileadmin/mevzuatek/otv_oranlari_tum/03072020_III_sayili_liste.pdf> | <https://www.statista.com/outlook/cmo/alcoholic-drinks/worldwide> |
| Turkmenistan | <http://minjust.gov.tm/mcenter-single-ru/38> | Information provided by the Ministry of Health, following and official request of the WHO |
| Ukraine | <https://www.profiwins.com.ua/ru/legislation/kodeks/1355.html> | <https://publikacije.stat.gov.rs/G2020/Pdf/G20202053.pdf> |
| United Kingdom | <https://ec.europa.eu/taxation_customs/business/excise-duties-alcohol-tobacco-energy/excise-duties-alcohol_en> | <https://www.statista.com/outlook/cmo/alcoholic-drinks/worldwide> |
| Uzbekistan | <https://www.profiwins.com.ua/ru/legislation/kodeks/1355.html> | <https://publikacije.stat.gov.rs/G2020/Pdf/G20202053.pdf> |

Supplementary Table S3. Avoidable new alcohol-attributable cancer cases and deaths by cancer site for different tax-increase scenarios for the entire WHO European Region.

| **Taxation increase scenario†** | **Cancer incidence** | | | **Cancer deaths** | | |
| --- | --- | --- | --- | --- | --- | --- |
|  | **Total number of avoided cancers** ‡ | **% alcohol-attributable cancers**§ | **% alcohol-related cancers**¶ | **Total number of avoided cancers deaths**‡ | **% alcohol-attributable cancers**§ | **% alcohol-related cancers**§§ |
| **Breast** |  |  |  |  |  |  |
| 20% | 735 (587-885) | 1·8 (1·7-1·9) | 0·1 (0·1-0·2) | 218 (174-264) | 1·8 (1·7-1·9) | 0·1 (0·1-0·2) |
| 50% | 1,836 (1,469-2,212) | 4·5 (4·2-4·7) | 0·3 (0·3-0·4) | 544 (434-660) | 4·6 (4·3-4·8) | 0·3 (0·3-0·4) |
| 100% | 3,670 (2,937-4,421) | 9·0 (8·5-9·5) | 0·7 (0·6-0·8) | 1,086 (868-1,318) | 9·2 (8·7-9·6) | 0·6 (0·5-0·8) |
| **Colorectum** |  |  |  |  |  |  |
| 20% | 696 (496-908) | 1·2 (1·1-1·3) | 0·1 (0·1-0·2) | 345 (246-450) | 1·2 (1·1-1·3) | 0·1 (0·1-0·1) |
| 50% | 1,757 (1,252-2,297) | 3·0 (2·8-3·2) | 0·3 (0·2-0·4) | 871 (620-1135) | 3·0 (2·9-3·3) | 0·3 (0·2-0·4) |
| 100% | 3,571 (2,542-4,675) | 6·0 (5·8-6·5) | 0·6 (0·4-0·8) | 1,770 (1,260-2,310) | 6·2 (5·9-6·7) | 0·6 (0·4-0·8) |
| **Larynx** |  |  |  |  |  |  |
| 20% | 123 (100-147) | 0·9 (0·8-1·0) | 0·3 (0·2-0·3) | 61 (49-72) | 0·9 (0·8-1·0) | 0·3 (0·2-0·3) |
| 50% | 313 (255-374) | 2·2 (2·0-2·5) | 0·6 (0·5-0·8) | 154 (125-184) | 2·2 (2·0-2·6) | 0·6 (0·5-0·8) |
| 100% | 643 (523-769) | 4·5 (4·0-5·1) | 1·3 (1·1-1·6) | 318 (257-380) | 4·6 (4·1-5·3) | 1·3 (1·1-1·6) |
| **Lip and oral cavity** |  |  |  |  |  |  |
| 20% | 237 (216-262) | 0·8 (0·8-0·9) | 0·4 (0·3-0·4) | 108 (99-120) | 0·8 (0·7-0·9) | 0·3 (0·3-0·4) |
| 50% | 602 (549-666) | 2·1 (1·9-2·3) | 0·9 (0·8-1·0) | 276 (251-307) | 2·0 (1·9-2·3) | 0·9 (0·8-1·0) |
| 100% | 1,238 (1,129-1,374) | 4·2 (3·9-4·7) | 1·8 (1·7-2·0) | 568 (516-633) | 4·2 (3·9-4·7) | 1·8 (1·7-2·0) |
| **Liver** |  |  |  |  |  |  |
| 20% | 63 (19-110) | 1·1 (1·0-1·2) | 0·1 (0·0-0·2) | 57 (17-100) | 1·1 (1·0-1·2) | 0·1 (0·0-0·2) |
| 50% | 159 (49-278) | 2·7 (2·6-2·9) | 0·2 (0·1-0·4) | 143 (44-254) | 2·7 (2·6-2·9) | 0·2 (0·1-0·4) |
| 100% | 324 (100-568) | 5·5 (5·2-5·9) | 0·5 (0·1-0·8) | 290 (89-516) | 5·5 (5·3-6·0) | 0·5 (0·1-0·8) |
| **Oesophagus**¶ |  |  |  |  |  |  |
| 20% | 98 (90-107) | 1·0 (0.9-1·0) | 0·2 (0·2-0·3) | 90 (83-99) | 1·0 (0·9-1·1) | 0·2 (0·2-0·3) |
| 50% | 249 (229-272) | 2·4 (2·3-2·7) | 0·6 (0·6-0·7) | 229 (211-250) | 2·5 (2·3-2·7) | 0·6 (0·6-0·7) |
| 100% | 509 (468-558) | 4.9 (4·6-5·4) | 1·2 (1·1-1·4) | 469 (431-513) | 5·1 (4·7-5·6) | 1·2 (1·2-1·4) |
| **Pharynx** |  |  |  |  |  |  |
| 20% | 145 (132-162) | 0·7 (0·6-0·8) | 0·3 (0·3-0·4) | 65 (59-73) | 0·7 (0·6-0·8) | 0·3 (0·3-0·4) |
| 50% | 369 (336-414) | 1·7 (1·6-2·0) | 0·9 (0·8-1·0) | 167 (151-186) | 1·7 (1·0·6-2) | 0·9 (0·8-1·0) |
| 100% | 762 (694-858) | 3·6 (3·3-4·2) | 1·8 (1·7-2·0) | 345 (312-386) | 3·6 (3·3-4·1) | 1·8 (1·6-2·0) |

† Tax increase on national, beverage-specific alcohol excise duties on beer, wine, and spirits.

‡ The numbers of avoided incident cancers and avoided deaths due to cancers have been rounded up; for this reason, the sum of these columns is not exactly equal to the results found in Table 2 of the main article.

§ Alcohol-attributable cancers refer to those cancer cases or deaths estimated to have been caused by alcohol.

§§ Alcohol-related cancers refer to all new cases or deaths for cancers whose risk is increased by alcohol consumption.

¶ In oesophagus cancer, only cases of squamous cell carcinoma were considered.

Supplementary Table S4. Avoidable new alcohol-attributable cancer cases and deaths for different tax-increase scenarios by countries of the WHO European Region.

| **Taxation increase scenario†** | **Cancer incidence** | | | **Cancer deaths** | | |
| --- | --- | --- | --- | --- | --- | --- |
|  | **Total number of avoided cancers**‡ | **% alcohol-attributable cancers**§ | **% alcohol-related cancers**¶ | **Total number of avoided cancers**‡ | **% alcohol-attributable cancers**§ | **% alcohol-related cancers**¶ |
| **Albania** |  |  |  |  |  |  |
| 20% | 1 (1-2) | 1·0 (0·8-1·2) | 0·1 (0·1-0·1) | 1 (1-1) | 0·9 (0·7-1·2) | 0·1 (0·1-0·1) |
| 50% | 4 (3-5) | 2·4 (1·9-2·9) | 0·2 (0·2-0·2) | 2 (2-3) | 2·4 (1·8-2·9) | 0·2 (0·1-0·2) |
| 100% | 8 (6-10) | 4·9 (3·9-5·9) | 0·4 (0·3-0·5) | 4 (3-6) | 4·8 (3·7-5·9) | 0·4 (0·3-0·5) |
| **Armenia** |  |  |  |  |  |  |
| 20% | 4 (3-6) | 3·4 (2·4-4·3) | 0·2 (0·1-0·2) | 3 (2-4) | 3·3 (2·4-4·2) | 0·2 (0·1-0·2) |
| 50% | 11 (7-15) | 8·4 (5·9-10·7) | 0·4 (0·3-0·5) | 7 (4-9) | 8·4 (5·9-10·7) | 0·4 (0·3-0·5) |
| 100% | 22 (15-30) | 16·9 (11·9-21·5) | 0·8 (0·5-1·1) | 13 (9-18) | 16·8 (11·8-21·4) | 0·8 (0·5-1·1) |
| **Austria** |  |  |  |  |  |  |
| 20% | 14 (12-17) | 0·7 (0·6-0·8) | 0·1 (0·1-0·1) | 6 (5-7) | 0·6 (0·6-0·7) | 0·1 (0·1-0·1) |
| 50% | 36 (29-43) | 1·7 (1·5-2·0) | 0·3 (0·2-0·3) | 15 (12-18) | 1·6 (1·4-1·9) | 0·2 (0·2-0·3) |
| 100% | 72 (59-87) | 3·4 (3·0-4·0) | 0·5 (0·4-0·6) | 30 (25-36) | 3·3 (2·8-3·8) | 0·5 (0·4-0·6) |
| **Azerbaijan** |  |  |  |  |  |  |
| 20% | 2 (1-2) | 1·4 (1·0-1·8) | 0·0 (0·0-0·0) | 1 (1-2) | 1·4 (1·0-1·8) | 0·0 (0·0-0·1) |
| 50% | 5 (3-6) | 3·5 (2·6-4·4) | 0·1 (0·1-0·1) | 3 (2-4) | 3·5 (2·6-4·4) | 0·1 (0·1-0·1) |
| 100% | 9 (7-12) | 7·0 (5·2-8·9) | 0·2 (0·1-0·2) | 7 (5-9) | 6·9 (5·2-8·9) | 0·2 (0·1-0·3) |
| **Belarus** |  |  |  |  |  |  |
| 20% | 23 (17-31) | 1·0 (0·8-1·3) | 0·2 (0·1-0·3) | 11 (9-15) | 0·9 (0·7-1·2) | 0·2 (0·1-0·3) |
| 50% | 58 (44-79) | 2·6 (2·0-3·4) | 0·5 (0·4-0·6) | 29 (22-39) | 2·2 (1·8-3·1) | 0·5 (0·4-0·7) |
| 100% | 119 (89-161) | 5·2 (4·0-6·9) | 1·0 (0·8-1·3) | 58 (45-80) | 4·6 (3·6-6·3) | 1·0 (0·8-1·4) |
| **Belgium** |  |  |  |  |  |  |
| 20% | 31 (25-38) | 1·0 (0·9-1·2) | 0·1 (0·1-0·2) | 13 (10-15) | 1·0 (0·9-1·2) | 0·1 (0·1-0·2) |
| 50% | 77 (63-96) | 2·6 (2·2-3·1) | 0·3 (0·3-0·4) | 32 (26-39) | 2·6 (2·2-3·1) | 0·3 (0·3-0·4) |
| 100% | 156 (128-194) | 5·3 (4·5-6·3) | 0·7 (0·6-0·9) | 64 (53-78) | 5·2 (4·4-6·2) | 0·7 (0·5-0·8) |
| **Bosnia and Herzegovina** | |  |  |  |  |  |
| 20% | 2 (1-2) | 0·5 (0·4-0·7) | 0·0 (0·0-0·0) | 1 (1-1) | 0·5 (0·4-0·6) | 0·0 (0·0-0·0) |
| 50% | 4 (3-5) | 1·4 (1·1-1·6) | 0·1 (0·1-0·1) | 3 (2-3) | 1·4 (1·1-1·6) | 0·1 (0·1-0·1) |
| 100% | 8 (6-10) | 2·8 (2·2-3·3) | 0·2 (0·1-0·2) | 5 (4-6) | 2·7 (2·2-3·3) | 0·2 (0·1-0·2) |
| **Bulgaria** |  |  |  |  |  |  |
| 20% | 18 (14-23) | 0·9 (0·8-1·2) | 0·1 (0·1-0·2) | 9 (7-11) | 0·9 (0·8-1·2) | 0·1 (0·1-0·2) |
| 50% | 45 (36-57) | 2·4 (2·0-2·9) | 0·3 (0·3-0·4) | 22 (18-28) | 2·4 (1·9-2·8) | 0·3 (0·3-0·4) |
| 100% | 91 (73-116) | 4·8 (4·0-6·0) | 0·7 (0·5-0·8) | 46 (37-58) | 4·6 (3·8-5·7) | 0·6 (0·5-0·8) |
| **Croatia** |  |  |  |  |  |  |
| 20% | 11 (9-14) | 0·8 (0·7-1·0) | 0·1 (0·1-0·2) | 5 (4-7) | 0·8 (0·6-0·9) | 0·1 (0·1-0·2) |
| 50% | 28 (23-35) | 2·1 (1·7-2·5) | 0·3 (0·3-0·4) | 13 (11-17) | 1·9 (1·6-2·4) | 0·3 (0·3-0·4) |
| 100% | 56 (46-70) | 4·2 (3·5-5·1) | 0·6 (0·5-0·8) | 27 (22-34) | 3·9 (3·3-4·9) | 0·6 (0·5-0·8) |
| **Cyprus** |  |  |  |  |  |  |
| 20% | 2 (1-2) | 0·7 (0·6-0·8) | 0·1 (0·1-0·1) | 0 (0-1) | 0·7 (0·6-0·8) | 0·1 (0·1-0·1) |
| 50% | 4 (3-5) | 1·8 (1·5-2·1) | 0·2 (0·2-0·3) | 1 (1-1) | 1·7 (1·4-2·0) | 0·2 (0·2-0·2) |
| 100% | 8 (6-10) | 3·6 (3·0-4·2) | 0·4 (0·3-0·5) | 2 (2-3) | 3·3 (2·8-4·0) | 0·4 (0·3-0·5) |
| **Czechia** |  |  |  |  |  |  |
| 20% | 32 (25-39) | 1·1 (1·0-1·3) | 0·2 (0·1-0·2) | 15 (12-19) | 1·0 (0·9-1·3) | 0·2 (0·1-0·2) |
| 50% | 80 (64-99) | 2·8 (2·4-3·4) | 0·5 (0·4-0·6) | 38 (30-47) | 2·6 (2·3-3·2) | 0·4 (0·4-0·6) |
| 100% | 162 (130-202) | 5·7 (4·9-6·9) | 0·9 (0·7-1·1) | 77 (61-97) | 5·4 (4·6-6·6) | 0·9 (0·7-1·1) |
| **Denmark** |  |  |  |  |  |  |
| 20% | 15 (13-19) | 1·0 (0·9-1·2) | 0·1 (0·1-0·2) | 6 (5-8) | 1·0 (0·9-1·2) | 0·1 (0·1-0·2) |
| 50% | 39 (32-47) | 2·6 (2·2-3·0) | 0·3 (0·3-0·4) | 16 (13-19) | 2·6 (2·2-3·0) | 0·3 (0·3-0·4) |
| 100% | 79 (64-95) | 5·2 (4·5-6·1) | 0·7 (0·5-0·8) | 32 (26-39) | 5·2 (4·4-6·2) | 0·6 (0·5-0·8) |
| **Estonia** |  |  |  |  |  |  |
| 20% | 5 (4-7) | 1·4 (1·2-1·7) | 0·2 (0·2-0·3) | 2 (2-3) | 1·3 (1·2-1·6) | 0·2 (0·2-0·3) |
| 50% | 14 (11-18) | 3·7 (3·2-4·3) | 0·6 (0·5-0·7) | 6 (5-7) | 3·4 (2·9-4·1) | 0·6 (0·5-0·7) |
| 100% | 29 (23-36) | 7·5 (6·5-8·9) | 1·2 (1·0-1·6) | 12 (10-15) | 7·0 (6·0-8·6) | 1·2 (1·0-1·5) |
| **Finland** |  |  |  |  |  |  |
| 20% | 26 (21-33) | 2·4 (2·0-3·0) | 0·3 (0·2-0·3) | 9 (7-12) | 2·3 (1·9-2·9) | 0·3 (0·2-0·3) |
| 50% | 66 (52-84) | 6·1 (5·1-7·5) | 0·7 (0·5-0·9) | 23 (18-30) | 5·9 (4·9-7·5) | 0·7 (0·5-0·8) |
| 100% | 135 (105-172) | 12·5 (10·3-15·2) | 1·4 (1·1-1·8) | 47 (37-61) | 12·1 (9·9-15·2) | 1·3 (1·0-1·7) |
| **France** |  |  |  |  |  |  |
| 20% | 156 (129-188) | 0·7 (0·6-0·8) | 0·1 (0·1-0·1) | 64 (53-77) | 0·7 (0·6-0·8) | 0·1 (0·1-0·1) |
| 50% | 391 (324-472) | 1·8 (1·6-2·1) | 0·3 (0·2-0·4) | 162 (133-195) | 1·8 (1·5-2·1) | 0·3 (0·2-0·3) |
| 100% | 790 (654-955) | 3·7 (3·2-4·3) | 0·6 (0·5-0·7) | 328 (269-395) | 3·6 (3·1-4·2) | 0·6 (0·5-0·7) |
| **Georgia** |  |  |  |  |  |  |
| 20% | 3 (2-3) | 0·7 (0·6-0·8) | 0·1 (0·1-0·1) | 2 (1-2) | 0·7 (0·5-0·8) | 0·1 (0·1-0·1) |
| 50% | 7 (6-9) | 1·7 (1·4-2·1) | 0·2 (0·1-0·2) | 4 (4-5) | 1·6 (1·3-2·0) | 0·2 (0·2-0·2) |
| 100% | 14 (11-17) | 3·5 (2·9-4·2) | 0·4 (0·3-0·5) | 9 (7-11) | 3·3 (2·7-4·1) | 0·4 (0·3-0·5) |
| **Germany** |  |  |  |  |  |  |
| 20% | 250 (199-307) | 1·0 (0·8-1·1) | 0·1 (0·1-0·2) | 104 (83-129) | 0·9 (0·8-1·1) | 0·1 (0·1-0·2) |
| 50% | 628 (500-772) | 2·4 (2·0-2·9) | 0·3 (0·3-0·4) | 261 (208-324) | 2·3 (1·9-2·8) | 0·3 (0·3-0·4) |
| 100% | 1,268 (1,008-1,560) | 4·9 (4·0-5·8) | 0·7 (0·6-0·9) | 529 (421-656) | 4·8 (3·9-5·7) | 0·7 (0·5-0·8) |
| **Greece** |  |  |  |  |  |  |
| 20% | 50 (40-64) | 2·5 (2·0-3·1) | 0·3 (0·2-0·3) | 21 (16-28) | 2·4 (2·0-3·1) | 0·2 (0·2-0·3) |
| 50% | 127 (100-161) | 6·3 (5·1-7·7) | 0·7 (0·5-0·8) | 54 (41-71) | 6·1 (4·9-7·8) | 0·6 (0·5-0·8) |
| 100% | 258 (203-330) | 12·8 (10·4-15·8) | 1·3 (1·0-1·7) | 110 (84-145) | 12·5 (10·1-16) | 1·3 (1·0-1·7) |
| **Hungary** |  |  |  |  |  |  |
| 20% | 47 (38-60) | 1·4 (1·2-1·7) | 0·2 (0·2-0·3) | 24 (20-32) | 1·3 (1·1-1·7) | 0·2 (0·2-0·3) |
| 50% | 118 (97-152) | 3·5 (2·9-4·4) | 0·6 (0·5-0·7) | 61 (51-80) | 3·3 (2·8-4·2) | 0·6 (0·5-0·7) |
| 100% | 241 (199-313) | 7·1 (6·0-8·9) | 1·2 (1·0-1·5) | 126 (103-166) | 6·8 (5·7-8·7) | 1·2 (1·0-1·5) |
| **Iceland** |  |  |  |  |  |  |
| 20% | 1 (1-1) | 2·8 (2·4-3·2) | 0·3 (0·2-0·3) | 0 (0-0) | 2·8 (2·4-3·2) | 0·2 (0·2-0·3) |
| 50% | 3 (2-3) | 7·0 (6·0-8·1) | 0·6 (0·5-0·8) | 1 (1-1) | 7·0 (6·0-8·1) | 0·6 (0·5-0·8) |
| 100% | 5 (4-6) | 14·3 (12·2-16·5) | 1·3 (1·0-1·6) | 2 (1-2) | 14·2 (12·1-16·4) | 1·3 (1·0-1·6) |
| **Ireland** |  |  |  |  |  |  |
| 20% | 19 (15-23) | 1·7 (1·5-2·0) | 0·2 (0·2-0·3) | 6 (5-8) | 1·6 (1·4-1·9) | 0·2 (0·2-0·3) |
| 50% | 47 (39-58) | 4·3 (3·7-5·0) | 0·6 (0·5-0·7) | 16 (13-20) | 4·1 (3·5-4·9) | 0·6 (0·5-0·7) |
| 100% | 95 (78-117) | 8·8 (7·5-10·3) | 1·2 (1·0-1·4) | 33 (27-41) | 8·4 (7·1-9·9) | 1·2 (0·9-1·4) |
| **Israel** |  |  |  |  |  |  |
| 20% | 3 (3-4) | 1·4 (1·1-1·6) | 0·0 (0·0-0·0) | 1 (1-2) | 1·4 (1·1-1·6) | 0·0 (0·0-0·0) |
| 50% | 9 (7-11) | 3·4 (2·8-4·0) | 0·1 (0·1-0·1) | 4 (3-5) | 3·4 (2·8-4·0) | 0·1 (0·1-0·1) |
| 100% | 17 (13-21) | 6·8 (5·7-8·0) | 0·2 (0·1-0·2) | 7 (6-9) | 6·8 (5·7-8·0) | 0·2 (0·1-0·2) |
| **Italy** |  |  |  |  |  |  |
| 20% | 130 (106-158) | 1·2 (1·0-1·4) | 0·1 (0·1-0·1) | 52 (42-63) | 1·2 (1·0-1·3) | 0·1 (0·1-0·1) |
| 50% | 327 (267-398) | 3·0 (2·5-3·4) | 0·2 (0·2-0·3) | 130 (105-158) | 3·0 (2·5-3·4) | 0·2 (0·2-0·3) |
| 100% | 658 (536-801) | 6·0 (5·1-6·9) | 0·5 (0·4-0·6) | 262 (211-319) | 5·9 (5·0-6·8) | 0·5 (0·4-0·6) |
| **Kazakhstan** |  |  |  |  |  |  |
| 20% | 14 (10-18) | 1·2 (1·0-1·7) | 0·1 (0·1-0·2) | 9 (7-11) | 1·2 (0·9-1·6) | 0·1 (0·1-0·2) |
| 50% | 35 (26-46) | 3·1 (2·4-4·2) | 0·3 (0·2-0·4) | 22 (17-29) | 3·0 (2·3-4·0) | 0·3 (0·2-0·4) |
| 100% | 71 (53-94) | 6·4 (4·9-8·4) | 0·6 (0·5-0·8) | 45 (34-59) | 6·0 (4·6-8·1) | 0·6 (0·5-0·8) |
| **Kyrgyzstan** |  |  |  |  |  |  |
| 20% | 3 (2-4) | 1·9 (1·5-2·6) | 0·2 (0·1-0·2) | 2 (1-2) | 1·8 (1·4-2·4) | 0·2 (0·1-0·2) |
| 50% | 7 (5-10) | 5·0 (3·8-6·8) | 0·4 (0·3-0·6) | 5 (4-6) | 4·6 (3·5-6·3) | 0·4 (0·3-0·6) |
| 100% | 15 (11-21) | 10·4 (8-14·5) | 0·9 (0·7-1·3) | 10 (7-13) | 9·7 (7·4-13·7) | 0·9 (0·7-1·2) |
| **Latvia** |  |  |  |  |  |  |
| 20% | 7 (5-9) | 1·7 (1·4-2·2) | 0·2 (0·2-0·3) | 4 (3-5) | 1·6 (1·3-2·0) | 0·2 (0·2-0·3) |
| 50% | 17 (14-22) | 4·4 (3·7-5·5) | 0·6 (0·5-0·8) | 10 (8-12) | 4·0 (3·3-5·2) | 0·6 (0·5-0·8) |
| 100% | 35 (28-45) | 9·0 (7·4-11·4) | 1·2 (1·0-1·6) | 20 (16-26) | 8·3 (6·7-10·8) | 1·2 (1·0-1·6) |
| **Lithuania** |  |  |  |  |  |  |
| 20% | 11 (9-14) | 1·6 (1·4-2·0) | 0·3 (0·2-0·4) | 6 (5-8) | 1·5 (1·2-1·8) | 0·3 (0·2-0·4) |
| 50% | 28 (23-35) | 4·2 (3·5-5·1) | 0·7 (0·6-0·9) | 16 (13-20) | 3·7 (3·1-4·7) | 0·7 (0·6-0·9) |
| 100% | 57 (47-73) | 8·5 (7·3-10·6) | 1·5 (1·2-1·9) | 33 (27-43) | 7·7 (6·4-9·9) | 1·5 (1·2-1·9) |
| **Luxembourg** |  |  |  |  |  |  |
| 20% | 1 (1-1) | 0·6 (0·5-0·7) | 0·1 (0·1-0·1) | 0 (0-0) | 0·5 (0·5-0·6) | 0·1 (0·1-0·1) |
| 50% | 2 (2-2) | 1·4 (1·2-1·6) | 0·2 (0·2-0·3) | 1 (1-1) | 1·4 (1·1-1·6) | 0·2 (0·2-0·3) |
| 100% | 4 (3-5) | 2·8 (2·3-3·3) | 0·4 (0·3-0·5) | 1 (1-2) | 2·7 (2·3-3·3) | 0·4 (0·3-0·5) |
| **Malta** |  |  |  |  |  |  |
| 20% | 1 (1-1) | 1·0 (0·8-1·2) | 0·1 (0·1-0·1) | 0 (0-0) | 1·0 (0·8-1·1) | 0·1 (0·1-0·1) |
| 50% | 2 (1-2) | 2·5 (2·1-2·9) | 0·2 (0·2-0·3) | 1 (1-1) | 2·4 (2·1-2·9) | 0·2 (0·2-0·3) |
| 100% | 3 (3-4) | 5·0 (4·3-5·9) | 0·4 (0·4-0·5) | 1 (1-2) | 4·9 (4·2-5·8) | 0·4 (0·4-0·5) |
| **Moldova** |  |  |  |  |  |  |
| 20% | 7 (4-8) | 1·4 (0·9-1·9) | 0·2 (0·1-0·2) | 4 (2-5) | 1·4 (0·8-1·8) | 0·2 (0·1-0·2) |
| 50% | 17 (9-20) | 3·6 (2·2-4·6) | 0·5 (0·2-0·5) | 11 (5-13) | 3·5 (2·0-4·5) | 0·5 (0·2-0·6) |
| 100% | 35 (18-41) | 7·4 (4·5-9·3) | 0·9 (0·5-1·1) | 22 (11-26) | 7·1 (4·1-9·1) | 1·0 (0·5-1·2) |
| **Montenegro** |  |  |  |  |  |  |
| 20% | 1 (1-2) | 1·4 (1·1-1·7) | 0·1 (0·1-0·2) | 1 (0-1) | 1·4 (1·0-1·7) | 0·1 (0·1-0·2) |
| 50% | 3 (2-4) | 3·6 (2·7-4·3) | 0·4 (0·3-0·4) | 2 (1-2) | 3·4 (2·6-4·2) | 0·4 (0·3-0·4) |
| 100% | 7 (5-8) | 7·2 (5·5-8·7) | 0·7 (0·5-0·9) | 3 (2-4) | 7·0 (5·3-8·5) | 0·7 (0·5-0·9) |
| **Netherlands** |  |  |  |  |  |  |
| 20% | 49 (40-60) | 1·1 (1·0-1·3) | 0·1 (0·1-0·1) | 18 (14-22) | 1·1 (1·0-1·3) | 0·1 (0·1-0·1) |
| 50% | 124 (101-151) | 2·9 (2·5-3·3) | 0·3 (0·3-0·4) | 44 (36-54) | 2·8 (2·4-3·3) | 0·3 (0·2-0·4) |
| 100% | 249 (204-304) | 5·8 (5·1-6·7) | 0·6 (0·5-0·7) | 89 (72-110) | 5·7 (4·9-6·6) | 0·6 (0·5-0·7) |
| **North Macedonia** |  |  |  |  |  |  |
| 20% | 2 (1-2) | 1·1 (0·8-1·2) | 0·1 (0·0-0·1) | 1 (1-1) | 1·1 (0·8-1·2) | 0·1 (0·0-0·1) |
| 50% | 4 (3-5) | 2·7 (2·0-3·1) | 0·1 (0·1-0·2) | 2 (2-3) | 2·7 (2·0-3·1) | 0·1 (0·1-0·2) |
| 100% | 8 (6-10) | 5·4 (4·0-6·2) | 0·3 (0·2-0·3) | 5 (3-6) | 5·4 (3·9-6·2) | 0·3 (0·2-0·4) |
| **Norway** |  |  |  |  |  |  |
| 20% | 32 (24-39) | 4·6 (3·8-5·4) | 0·4 (0·3-0·5) | 12 (9-15) | 4·7 (3·8-5·5) | 0·4 (0·3-0·4) |
| 50% | 80 (60-99) | 11·7 (9·7-13·7) | 0·9 (0·7-1·2) | 30 (21-37) | 11·7 (9·7-13·8) | 0·9 (0·6-1·1) |
| 100% | 162 (120-202) | 23·7 (19·8-27·6) | 1·9 (1·4-2·3) | 60 (43-76) | 23·8 (19·7-27·9) | 1·8 (1·3-2·3) |
| **Poland** |  |  |  |  |  |  |
| 20% | 135 (108-171) | 1·7 (1·4-2·1) | 0·3 (0·2-0·3) | 81 (65-106) | 1·6 (1·3-2·1) | 0·2 (0·2-0·3) |
| 50% | 342 (274-435) | 4·2 (3·5-5·3) | 0·6 (0·5-0·8) | 206 (164-269) | 4·1 (3·4-5·2) | 0·6 (0·5-0·8) |
| 100% | 700 (559-895) | 8·7 (7·2-10·9) | 1·3 (1·1-1·7) | 422 (337-555) | 8·4 (6·9-10·8) | 1·3 (1·0-1·7) |
| **Portugal** |  |  |  |  |  |  |
| 20% | 32 (27-40) | 0·9 (0·8-1·1) | 0·1 (0·1-0·2) | 14 (11-17) | 0·9 (0·7-1·0) | 0·1 (0·1-0·2) |
| 50% | 82 (67-100) | 2·3 (1·9-2·7) | 0·4 (0·3-0·5) | 36 (29-44) | 2·2 (1·8-2·6) | 0·4 (0·3-0·4) |
| 100% | 166 (137-204) | 4·6 (3·9-5·5) | 0·8 (0·6-0·9) | 73 (59-90) | 4·4 (3·7-5·4) | 0·7 (0·6-0·9) |
| **Romania** |  |  |  |  |  |  |
| 20% | 60 (46-76) | 1·1 (0·9-1·5) | 0·2 (0·2-0·3) | 34 (25-42) | 1·1 (0·8-1·4) | 0·2 (0·2-0·3) |
| 50% | 153 (116-193) | 2·9 (2·2-3·7) | 0·5 (0·4-0·7) | 85 (64-107) | 2·8 (2·1-3·6) | 0·5 (0·4-0·7) |
| 100% | 311 (236-395) | 5·9 (4·5-7·6) | 1·1 (0·8-1·4) | 173 (130-219) | 5·6 (4·2-7·4) | 1·1 (0·8-1·4) |
| **Russia** |  |  |  |  |  |  |
| 20% | 276 (213-354) | 1·1 (0·8-1·4) | 0·2 (0·1-0·2) | 141 (110-181) | 1·0 (0·8-1·3) | 0·2 (0·1-0·2) |
| 50% | 696 (537-894) | 2·7 (2·1-3·4) | 0·4 (0·3-0·5) | 356 (277-459) | 2·5 (1·9-3·3) | 0·4 (0·3-0·5) |
| 100% | 1,414 (1,090-1,819) | 5·4 (4·3-7·0) | 0·8 (0·6-1·0) | 727 (561-938) | 5·0 (3·9-6·7) | 0·8 (0·6-1·0) |
| **Serbia** |  |  |  |  |  |  |
| 20% | 17 (13-21) | 0·9 (0·7-1·1) | 0·1 (0·1-0·1) | 9 (7-11) | 0·8 (0·7-1·1) | 0·1 (0·1-0·1) |
| 50% | 42 (34-53) | 2·2 (1·8-2·7) | 0·3 (0·2-0·3) | 22 (17-28) | 2·1 (1·7-2·7) | 0·3 (0·2-0·3) |
| 100% | 85 (68-107) | 4·3 (3·6-5·4) | 0·5 (0·4-0·7) | 44 (35-56) | 4·2 (3·4-5·4) | 0·5 (0·4-0·6) |
| **Slovakia** |  |  |  |  |  |  |
| 20% | 21 (17-27) | 1·4 (1·1-1·7) | 0·2 (0·2-0·3) | 10 (8-13) | 1·3 (1·0-1·6) | 0·2 (0·2-0·3) |
| 50% | 54 (43-70) | 3·4 (2·9-4·3) | 0·5 (0·4-0·7) | 26 (21-34) | 3·2 (2·6-4·1) | 0·6 (0·4-0·7) |
| 100% | 109 (87-142) | 7·0 (5·8-8·9) | 1·1 (0·9-1·4) | 54 (43-70) | 6·6 (5·4-8·5) | 1·1 (0·9-1·5) |
| **Slovenia** |  |  |  |  |  |  |
| 20% | 6 (5-7) | 1·1 (0·9-1·2) | 0·2 (0·1-0·2) | 3 (2-3) | 1·0 (0·9-1·2) | 0·1 (0·1-0·2) |
| 50% | 15 (12-18) | 2·7 (2·3-3·1) | 0·4 (0·3-0·5) | 7 (6-9) | 2·6 (2·2-3·1) | 0·4 (0·3-0·5) |
| 100% | 30 (25-37) | 5·4 (4·6-6·4) | 0·8 (0·7-1·0) | 14 (11-18) | 5·3 (4·5-6·4) | 0·8 (0·6-0·9) |
| **Spain** |  |  |  |  |  |  |
| 20% | 84 (69-104) | 0·6 (0·5-0·8) | 0·1 (0·1-0·1) | 31 (25-39) | 0·6 (0·5-0·8) | 0·1 (0·1-0·1) |
| 50% | 211 (173-260) | 1·6 (1·3-1·9) | 0·2 (0·2-0·3) | 79 (64-99) | 1·6 (1·3-1·9) | 0·2 (0·2-0·3) |
| 100% | 425 (349-525) | 3·2 (2·7-3·8) | 0·4 (0·4-0·5) | 159 (128-199) | 3·2 (2·7-3·9) | 0·4 (0·3-0·5) |
| **Sweden** |  |  |  |  |  |  |
| 20% | 26 (20-32) | 1·9 (1·5-2·2) | 0·2 (0·1-0·2) | 10 (8-13) | 1·8 (1·5-2·2) | 0·1 (0·1-0·2) |
| 50% | 64 (50-79) | 4·7 (3·9-5·5) | 0·4 (0·3-0·5) | 25 (19-33) | 4·6 (3·8-5·5) | 0·4 (0·3-0·5) |
| 100% | 130 (101-160) | 9·4 (7·8-11·0) | 0·8 (0·6-0·9) | 51 (39-66) | 9·3 (7·6-11·1) | 0·8 (0·6-1·0) |
| **Switzerland** |  |  |  |  |  |  |
| 20% | 16 (13-20) | 0·9 (0·7-1·0) | 0·1 (0·1-0·1) | 6 (5-7) | 0·8 (0·7-1·0) | 0·1 (0·1-0·1) |
| 50% | 41 (32-50) | 2·2 (1·8-2·5) | 0·3 (0·2-0·3) | 15 (12-19) | 2·1 (1·8-2·5) | 0·3 (0·2-0·3) |
| 100% | 82 (65-100) | 4·4 (3·7-5·1) | 0·6 (0·4-0·7) | 31 (24-38) | 4·2 (3·6-5·0) | 0·6 (0·4-0·7) |
| **Tajikistan** |  |  |  |  |  |  |
| 20% | 0 (0-1) | 2·3 (1·8-2·6) | 0·0 (0·0-0·0) | 0 (0-1) | 2·3 (1·7-2·6) | 0·0 (0·0-0·0) |
| 50% | 1 (1-2) | 5·8 (4·4-6·5) | 0·1 (0·0-0·1) | 1 (0-1) | 5·8 (4·4-6·5) | 0·1 (0·0-0·1) |
| 100% | 2 (1-3) | 11·5 (8·8-12·9) | 0·1 (0·1-0·2) | 2 (1-3) | 11·6 (8·7-12·9) | 0·1 (0·1-0·2) |
| **Turkmenistan** |  |  |  |  |  |  |
| 20% | 14 (12-18) | 1·9 (1·6-2·3) | 0·0 (0·0-0·0) | 7 (6-9) | 1·8 (1·5-2·2) | 0·0 (0·0-0·0) |
| 50% | 36 (29-47) | 4·8 (4·1-5·8) | 0·1 (0·1-0·1) | 18 (15-24) | 4·6 (3·9-5·6) | 0·1 (0·1-0·1) |
| 100% | 75 (61-97) | 9·8 (8·3-12) | 0·2 (0·1-0·2) | 38 (31-49) | 9·4 (8·0-11·6) | 0·2 (0·1-0·2) |
| **Turkey** |  |  |  |  |  |  |
| 20% | 2 (2-3) | 2·0 (1·6-2·6) | 0·1 (0·1-0·1) | 2 (1-2) | 2·0 (1·5-2·5) | 0·1 (0·1-0·1) |
| 50% | 6 (4-7) | 5·2 (4·0-6·4) | 0·3 (0·2-0·4) | 4 (3-5) | 5·1 (3·8-6·3) | 0·3 (0·2-0·4) |
| 100% | 11 (9-14) | 10·6 (8·2-13·1) | 0·6 (0·5-0·7) | 8 (6-10) | 10·3 (7·9-12·9) | 0·6 (0·5-0·8) |
| **Ukraine** |  |  |  |  |  |  |
| 20% | 81 (58-98) | 1·0 (0·7-1·3) | 0·1 (0·1-0·2) | 48 (34-59) | 0·9 (0·6-1·2) | 0·1 (0·1-0·2) |
| 50% | 204 (147-248) | 2·4 (1·7-3·2) | 0·4 (0·3-0·5) | 122 (87-150) | 2·3 (1·6-3·1) | 0·4 (0·3-0·5) |
| 100% | 413 (297-505) | 4·9 (3·5-6·4) | 0·8 (0·6-0·9) | 248 (176-304) | 4·7 (3·3-6·3) | 0·8 (0·5-0·9) |
| **United Kingdom** |  |  |  |  |  |  |
| 20% | 353 (291-438) | 2·1 (1·8-2·6) | 0·3 (0·2-0·3) | 132 (108-166) | 2·1 (1·8-2·6) | 0·3 (0·2-0·3) |
| 50% | 891 (736-1,107) | 5·4 (4·6-6·5) | 0·7 (0·6-0·9) | 334 (274-421) | 5·3 (4·6-6·6) | 0·6 (0·5-0·8) |
| 100% | 1,813 (1,496-2,262) | 10·9 (9·4-13·3) | 1·4 (1·2-1·7) | 681 (558-864) | 10·9 (9·3-13·6) | 1·3 (1·1-1·7) |
| **Uzbekistan** |  |  |  |  |  |  |
| 20% | 1 (0-1) | 0·1 (0·1-0·2) | 0·0 (0·0-0·0) | 0 (0-0) | 0·1 (0·1-0·2) | 0·0 (0·0-0·0) |
| 50% | 1 (1-2) | 0·3 (0·3-0·4) | 0·0 (0·0-0·0) | 1 (1-1) | 0·3 (0·3-0·4) | 0·0 (0·0-0·0) |
| 100% | 3 (2-3) | 0·7 (0·5-0·9) | 0·0 (0·0-0·0) | 2 (1-2) | 0·7 (0·5-0·8) | 0·0 (0·0-0·0) |

† Tax increase on national, beverage-specific alcohol excise duties on beer, wine, and spirits.

‡ The numbers of avoided incident cancers and avoided deaths due to cancers have been rounded up; for this reason, the sum of these columns is not exactly equal to the results found in Table 2 of the main article.

§ Alcohol-attributable cancers refer to those cancer cases or deaths estimated to have been caused by alcohol.

¶ Alcohol-related cancers refer to all new cases or deaths for cancers whose risk is increased by alcohol consumption.

Supplementary Table S5. Sensitivity analysis 1: Avoidable new alcohol-attributable cancer cases and deaths for applying the same tax rate as Finland, entire WHO European Region.

| **Cancer site** | **Cancer incidence** | | | **Cancer deaths** | | |
| --- | --- | --- | --- | --- | --- | --- |
|  | **Total number of avoided cancers**† | **% alcohol-attributable cancers**‡ | **% alcohol-related cancers**§ | **Total number of avoided cancers**† | **% alcohol-attributable cancers**‡ | **% alcohol-related cancers**§ |
| Breast | 3,057 (2,435-3,690) | 7·5 (7·1-7·9) | 0·6 (0·5-0·7) | 894 (709-1,085) | 7·5 (7·1-8·0) | 0·5 (0·4-0·6) |
| Colorectum | 3,061 (2,206-4,010) | 5·2 (4·9-5·6) | 0·5 (0·4-0·7) | 1,466 (1,051-1,919) | 5·1 (4·9-5·5) | 0·5 (0·3-0·6) |
| Larynx | 553 (450-654) | 3·8 (3·5-4·4) | 1·1 (0·9-1·3) | 267 (216-316) | 3·8 (3·4-4·4) | 1·1 (0·9-1·3) |
| Lip and oral cavity | 1,061 (969-1,173) | 3·6 (3·4-4·0) | 1·6 (1·4-1·7) | 462 (418-513) | 3·4 (3·1-3·8) | 1·5 (1·3-1·6) |
| Liver | 305 (93-531) | 5·2 (4·9-5·6) | 0·4 (0·1-0·8) | 274 (83-480) | 5·2 (4·9-5·6) | 0·4 (0·1-0·8) |
| Oesophagus¶ | 431 (394-470) | 4·2 (3·9-4·6) | 1·1 (1·0-1·1) | 382 (350-414) | 4·1 (3·8-4·5) | 1·0 (0·9-1·1) |
| Pharynx | 657 (599-736) | 3·1 (2·9-3·6) | 1·6 (1·4-1·7) | 308 (279-341) | 3·2 (2·9-3·6) | 1·6 (1·5-1·8) |
| Total | 9,123 (7,975-10,389) | 5·0 (4·8-5·4) | 0·7 (0·6-0·7) | 4,051 (3,525-4,633) | 4·8 (4·5-5·1) | 0·6 (0·5-0·7) |

† The numbers of avoided incident cancers and avoided deaths due to cancers have been rounded up; for this reason, the sum of these columns is not exactly equal to the total values shown in the main article.

‡ Alcohol-attributable cancers refer to those cancer cases or deaths estimated to have been caused by alcohol.

§ Alcohol-related cancers refer to all new cases or deaths for cancers whose risk is increased by alcohol consumption.

¶ In oesophagus cancer, only cases of squamous cell carcinoma were considered.

Supplementary Table S6. Sensitivity analysis 1: Avoidable new alcohol-attributable cancer cases and deaths for applying the same tax rate as Finland, by countries of the WHO European Region.

| **Country** | **Cancer incidence** | | | **Cancer deaths** | | |
| --- | --- | --- | --- | --- | --- | --- |
|  | **Total number of avoided cancers**† | **% alcohol-attributable cancers**‡ | **% alcohol-related cancers**§ | **Total number of avoided cancers**† | **% alcohol-attributable cancers**‡ | **% alcohol-related cancers**§ |
| Albania | 12 (10-15) | 7·8 (6·4-9·4) | 0·6 (0·5-0·8) | 7 (6-9) | 7·6 (6·1-9·4) | 0·6 (0·5-0·8) |
| Armenia | -3 (-5--1) | -2·4 (-3·6--1·2) | -0·1 (-0·2--0·1) | -2 (-3--1) | -2·4 (-3·5--1·2) | -0·1 (-0·2--0·1) |
| Austria | 161 (130-195) | 7·6 (6·4-9·0) | 1·1 (0·9-1·4) | 67 (54-81) | 7·3 (6·1-8·6) | 1·1 (0·9-1·3) |
| Azerbaijan | 12 (9-15) | 8·8 (7·2-10·5) | 0·2 (0·2-0·3) | 8 (6-11) | 8·8 (7·2-10·5) | 0·2 (0·2-0·3) |
| Belarus | 70 (54-88) | 3·0 (2·5-3·8) | 0·6 (0·5-0·7) | 34 (27-44) | 2·7 (2·2-3·5) | 0·6 (0·5-0·7) |
| Belgium | 165 (131-206) | 5·6 (4·6-6·7) | 0·7 (0·6-0·9) | 67 (54-84) | 5·5 (4·5-6·6) | 0·7 (0·6-0·9) |
| Bosnia and Herzegovina | 29 (20-38) | 9·7 (7·4-12·4) | 0·6 (0·4-0·8) | 18 (13-24) | 9·6 (7·2-12·4) | 0·6 (0·4-0·8) |
| Bulgaria | 115 (94-142) | 6·1 (5·1-7·4) | 0·8 (0·7-1·0) | 57 (46-71) | 5·8 (4·9-7·1) | 0·8 (0·7-1·0) |
| Croatia | 78 (64-101) | 5·9 (5·0-7·3) | 0·9 (0·7-1·1) | 38 (31-49) | 5·5 (4·6-7·1) | 0·9 (0·7-1·1) |
| Cyprus | 20 (16-25) | 9·3 (8·0-10·8) | 1·1 (0·9-1·3) | 6 (5-8) | 8·7 (7·4-10·2) | 1·0 (0·8-1·3) |
| Czechia | 139 (109-179) | 4·9 (4·0-6·1) | 0·8 (0·6-1·0) | 66 (51-86) | 4·6 (3·8-5·9) | 0·8 (0·6-1·0) |
| Denmark | 104 (85-129) | 6·9 (5·8-8·3) | 0·9 (0·7-1·1) | 42 (34-53) | 6·9 (5·8-8·3) | 0·8 (0·7-1·0) |
| Estonia | 3 (2-4) | 0·7 (0·6-0·9) | 0·1 (0·1-0·2) | 1 (1-2) | 0·7 (0·6-0·8) | 0·1 (0·1-0·1) |
| Finland | 0 (0-0) | 0·0 (0·0-0·0) | 0·0 (0·0-0·0) | 0 (0-0) | 0·0 (0·0-0·0) | 0·0 (0·0-0·0) |
| France | 1,183 (967-1,441) | 5·5 (4·7-6·4) | 0·9 (0·7-1·1) | 491 (404-598) | 5·4 (4·6-6·3) | 0·8 (0·7-1·0) |
| Georgia | 32 (26-40) | 7·9 (6·6-9·5) | 0·8 (0·7-1·0) | 20 (16-24) | 7·5 (6·2-9·2) | 0·9 (0·7-1·0) |
| Germany | 1,694 (1,322-2,137) | 6·6 (5·3-8·0) | 0·9 (0·7-1·2) | 708 (555-905) | 6·4 (5·1-7·8) | 0·9 (0·7-1·1) |
| Greece | 57 (41-76) | 2·8 (2·1-3·7) | 0·3 (0·2-0·4) | 25 (17-33) | 2·8 (2·0-3·7) | 0·3 (0·2-0·4) |
| Hungary | 167 (136-212) | 4·9 (4·1-6·1) | 0·8 (0·7-1·1) | 87 (71-113) | 4·7 (3·9-6·0) | 0·8 (0·7-1·1) |
| Iceland | 0 (0-1) | 1·3 (0·8-1·8) | 0·1 (0·1-0·2) | 0 (0-0) | 1·3 (0·8-1·8) | 0·1 (0·1-0·2) |
| Ireland | 36 (28-47) | 3·3 (2·7-4·0) | 0·5 (0·4-0·6) | 12 (10-16) | 3·2 (2·5-3·9) | 0·4 (0·3-0·6) |
| Israel | 31 (25-39) | 12·5 (10·7-14·3) | 0·3 (0·3-0·4) | 13 (10-17) | 12·5 (10·7-14·3) | 0·3 (0·3-0·4) |
| Italy | 817 (669-995) | 7·5 (6·4-8·6) | 0·6 (0·5-0·7) | 325 (263-397) | 7·4 (6·3-8·5) | 0·6 (0·5-0·8) |
| Kazakhstan | 47 (39-57) | 4·2 (3·5-5·2) | 0·4 (0·3-0·5) | 30 (25-36) | 4·0 (3·3-5·0) | 0·4 (0·4-0·5) |
| Kyrgyzstan | -5 (-7--4) | -3·4 (-4·8--2·5) | -0·3 (-0·4--0·2) | -3 (-4--2) | -3·1 (-4·5--2·3) | -0·3 (-0·4--0·2) |
| Latvia | 5 (4-7) | 1·4 (1·1-1·8) | 0·2 (0·1-0·3) | 3 (2-4) | 1·3 (1·0-1·7) | 0·2 (0·1-0·3) |
| Lithuania | 4 (1-7) | 0·6 (0·1-1·1) | 0·1 (0·0-0·2) | 2 (0-4) | 0·5 (0·1-1·0) | 0·1 (0·0-0·2) |
| Luxembourg | 13 (11-16) | 9·6 (8·2-11·2) | 1·4 (1·2-1·7) | 5 (4-6) | 9·4 (8·1-11·1) | 1·4 (1·1-1·7) |
| Malta | 7 (6-8) | 10·7 (9·2-12·2) | 0·9 (0·8-1·1) | 3 (2-3) | 10·5 (9·1-12·1) | 0·9 (0·8-1·2) |
| Moldova | 32 (15-37) | 6·7 (3·8-8·4) | 0·9 (0·4-1·0) | 20 (9-23) | 6·4 (3·4-8·2) | 0·9 (0·4-1·1) |
| Montenegro | 5 (4-6) | 5·4 (4·2-6·4) | 0·5 (0·4-0·6) | 3 (2-3) | 5·2 (3·9-6·3) | 0·6 (0·4-0·7) |
| Netherlands | 299 (238-376) | 7·0 (5·8-8·3) | 0·7 (0·6-0·9) | 107 (86-135) | 6·8 (5·7-8·2) | 0·7 (0·6-0·9) |
| North Macedonia | 20 (13-24) | 13·0 (10·0-15·0) | 0·7 (0·5-0·8) | 12 (8-15) | 12·9 (9·7-14·9) | 0·7 (0·5-0·9) |
| Norway | -49 (-61--37) | -7·2 (-8·4--5·8) | -0·6 (-0·7--0·4) | -18 (-23--13) | -7·2 (-8·6--5·8) | -0·5 (-0·7--0·4) |
| Poland | 127 (72-190) | 1·6 (0·9-2·3) | 0·2 (0·1-0·4) | 76 (43-115) | 1·5 (0·9-2·2) | 0·2 (0·1-0·3) |
| Portugal | 156 (128-197) | 4·3 (3·6-5·3) | 0·7 (0·6-0·9) | 68 (54-88) | 4·1 (3·4-5·2) | 0·7 (0·5-0·9) |
| Romania | 256 (193-320) | 4·8 (3·7-6·2) | 0·9 (0·7-1·1) | 143 (107-178) | 4·6 (3·5-6·1) | 0·9 (0·7-1·1) |
| Russia | 906 (716-1,121) | 3·5 (2·8-4·3) | 0·5 (0·4-0·6) | 465 (367-575) | 3·2 (2·6-4·1) | 0·5 (0·4-0·6) |
| Serbia | 201 (158-257) | 10·3 (8·4-12·9) | 1·2 (1·0-1·6) | 105 (82-135) | 10·0 (8·0-12·9) | 1·2 (1·0-1·6) |
| Slovakia | 69 (55-90) | 4·4 (3·6-5·7) | 0·7 (0·6-0·9) | 34 (27-45) | 4·1 (3·3-5·5) | 0·7 (0·6-0·9) |
| Slovenian | 30 (24-38) | 5·4 (4·5-6·6) | 0·8 (0·7-1·0) | 14 (11-18) | 5·4 (4·4-6·5) | 0·8 (0·6-1·0) |
| Spain | 1,039 (831-1,323) | 7·9 (6·5-9·7) | 1·1 (0·9-1·4) | 389 (305-499) | 7·9 (6·5-9·8) | 1·0 (0·8-1·3) |
| Sweden | 83 (64-103) | 6·0 (5·0-7·1) | 0·5 (0·4-0·6) | 33 (24-42) | 5·9 (4·9-7·1) | 0·5 (0·4-0·6) |
| Switzerland | 164 (133-196) | 8·7 (7·5-10·0) | 1·1 (0·9-1·3) | 62 (50-74) | 8·5 (7·5-10·0) | 1·1 (0·9-1·3) |
| Tajikistan | 1 (1-2) | 7·4 (5·4-8·4) | 0·1 (0·0-0·1) | 1 (1-2) | 7·4 (5·4-8·4) | 0·1 (0·0-0·1) |
| Turkmenistan | 1 (0-1) | 0·7 (0·3-1·1) | 0·0 (0·0-0·1) | 1 (0-1) | 0·7 (0·3-1·1) | 0·0 (0·0-0·1) |
| Turkey | -2 (-3--1) | -0·2 (-0·4--0·1) | 0·0 (0·0-0·0) | -1 (-2-0) | -0·2 (-0·4--0·1) | 0·0 (0·0-0·0) |
| Ukraine | 427 (304-516) | 5·0 (3·6-6·5) | 0·8 (0·6-0·9) | 256 (182-308) | 4·9 (3·4-6·3) | 0·8 (0·6-0·9) |
| United Kingdom | 311 (215-428) | 1·9 (1·3-2·5) | 0·2 (0·2-0·3) | 117 (81-161) | 1·9 (1·3-2·5) | 0·2 (0·2-0·3) |
| Uzbekistan | 54 (42-67) | 13·4 (10·8-16·5) | 0·4 (0·3-0·6) | 36 (28-46) | 13·1 (10·4-16·4) | 0·5 (0·4-0·6) |

† The numbers of avoided incident cancers and avoided deaths due to cancers have been rounded up; for this reason, the sum of these columns is not exactly equal to the total values shown in the main article. In countries with higher alcohol excise taxes on beer, wine or spirits than in Finland, the numbers of avoidable new cancer cases and deaths may be lower than 0 if the relevant alcoholic beverages account for a relatively high share of per capita consumption (i.e., Armenia, Kyrgyzstan, Norway and Turkey).

‡ Alcohol-attributable cancers refer to those cancer cases or deaths estimated to have been caused by alcohol.

§ Alcohol-related cancers refer to all new cases or deaths for cancers whose risk is increased by alcohol consumption.

Supplementary Table S7. Sensitivity analysis 2: Avoidable new alcohol-attributable cancer cases and deaths for each tax increase scenario by cancer site and for the entire WHO European Region, applying a lag time of 20 years between alcohol exposure and cancer development and deaths.

| **Taxation increase scenario†** | **Cancer incidence** | | | **Cancer deaths** | | |
| --- | --- | --- | --- | --- | --- | --- |
|  | **Total number of avoided cancers** ‡ | **% alcohol-attributable cancers**§ | **% alcohol-related cancers**¶ | **Total number of avoided cancers deaths**‡ | **% alcohol-attributable cancers**§ | **% alcohol-related cancers**§§ |
| **Breast** |  |  |  |  |  |  |
| 20% | 707 (562-849) | 1·7 (1·6-1·8) | 0·1 (0·1-0·2) | 204 (163-245) | 1·8 (1·7-1·9) | 0·1 (0·1-0·1) |
| 50% | 1,769 (1,406-2,122) | 4·3 (4·0-4·5) | 0·3 (0·3-0·4) | 510 (406-613) | 4·4 (4·1-4·6) | 0·3 (0·2-0·4) |
| 100% | 3,537 (2,813-4,244) | 8·5 (8·1-9·1) | 0·7 (0·5-0·8) | 1,019 (813-1,225) | 8·7 (8·3-9·3) | 0·6 (0·5-0·7) |
| **Colorectum** |  |  |  |  |  |  |
| 20% | 664 (446-858) | 1·1 (1·1-1·2) | 0·1 (0·1-0·1) | 325 (216-421) | 1·2 (1·1-1·2) | 0·1 (0·1-0·1) |
| 50% | 1,676 (1,126-2,166) | 2·8 (2·7-3·0) | 0·3 (0·2-0·4) | 820 (544-1064) | 2·9 (2·7-3·1) | 0·3 (0·2-0·3) |
| 100% | 3,405 (2,287-4,404) | 5·7 (5·4-6·2) | 0·6 (0·4-0·7) | 1,667 (1,106-2,163) | 5·9 (5·6-6·4) | 0·5 (0·4-0·7) |
| **Larynx** |  |  |  |  |  |  |
| 20% | 118 (96-138) | 0·8 (0·8-1·0) | 0·2 (0·2-0·3) | 58 (47-69) | 0·9 (0·8-1·0) | 0·2 (0·2-0·3) |
| 50% | 299 (244-351) | 2·1 (1·9-2·4) | 0·6 (0·5-0·7) | 148 (120-175) | 2·2 (2·0-2·5) | 0·6 (0·5-0·7) |
| 100% | 614 (501-723) | 4·4 (3·9-5·0) | 1·3 (1·0-1·5) | 304 (246-361) | 4·6 (4·0-5·2) | 1·3 (1·0-1·5) |
| **Lip and oral cavity** |  |  |  |  |  |  |
| 20% | 232 (207-255) | 0·8 (0·7-0·9) | 0·3 (0·3-0·4) | 107 (94-118) | 0·8 (0·7-0·9) | 0·3 (0·3-0·4) |
| 50% | 590 (525-649) | 2·0 (1·9-2·3) | 0·9 (0·8-1·0) | 272 (239-301) | 2·1 (1·9-2·3) | 0·9 (0·8-1·0) |
| 100% | 1,212 (1,077-1,335) | 4·2 (3·9-4·7) | 1·8 (1·6-2·0) | 559 (491-620) | 4·3 (3·9-4·8) | 1·8 (1·6-2·0) |
| **Liver** |  |  |  |  |  |  |
| 20% | 60 (17-102) | 1·0 (0·9-1·1) | 0·1 (0·0-0·1) | 54 (16-91) | 1·0 (1·0-1·1) | 0·1 (0·0-0·1) |
| 50% | 152 (44-259) | 2·5 (2·4-2·7) | 0·2 (0·1-0·4) | 136 (40-230) | 2·6 (2·4-2·8) | 0·2 (0·1-0·4) |
| 100% | 309 (89-528) | 5·1 (4·8-5·5) | 0·5 (0·1-0·8) | 277 (80-469) | 5·2 (4·9-5·6) | 0·4 (0·1-0·7) |
| **Oesophagus**¶ |  |  |  |  |  |  |
| 20% | 94 (86-102) | 0·9 (0·9-1·0) | 0·2 (0·2-0·2) | 86 (79-94) | 1·0 (0·9-1·1) | 0·2 (0·2-0·3) |
| 50% | 238 (217-260) | 2·4 (2·2-2·6) | 0·6 (0·5-0·6) | 219 (199-239) | 2·4 (2·2-2·7) | 0·6 (0·5-0·6) |
| 100% | 487 (444-534) | 4·8 (4·5-5·3) | 1·2 (1·1-1·3) | 447 (407-490) | 5·0 (4·6-5·5) | 1·2 (1·1-1·3) |
| **Pharynx** |  |  |  |  |  |  |
| 20% | 141 (126-156) | 0·7 (0·6-0·8) | 0·3 (0·3-0·4) | 65 (58-72) | 0·7 (0·6-0·8) | 0·3 (0·3-0·4) |
| 50% | 360 (321-399) | 1·7 (1·6-2·0) | 0·9 (0·8-1·0) | 166 (147-183) | 1·8 (1·6-2·0) | 0·9 (0·8-0·9) |
| 100% | 742 (662-827) | 3·6 (3·2-4·1) | 1·8 (1·6-2·0) | 343 (303-377) | 3·7 (3·3-4·1) | 1·8 (1·6-2·0) |
| **Total** |  |  |  |  |  |  |
| 20% | 2,017 (1,727-2,284) | 1·1 (1·1-1·2) | 0·1 (0·1-0·2) | 900 (762-1,024) | 1·1 (1·0-1·2) | 0·1 (0·1-0·2) |
| 50% | 5,084 (4,349-5,761) | 2·8 (2·6-3·0) | 0·4 (0·3-0·4) | 2,271 (1,923-2,588) | 2·7 (2·6-2·9) | 0·3 (0·3-0·4) |
| 100% | 10,308 (8,810-11,676) | 5·7 (5·4-6·1) | 0·7 (0·6-0·8) | 4,616 (3,911-5,264) | 5·6 (5·2-6·0) | 0·7 (0·6-0·8) |

† The numbers of avoided incident cancers and avoided deaths due to cancers have been rounded up; for this reason, the sum of these columns is not exactly equal to the total values shown in the main article.

‡ Alcohol-attributable cancers refer to those cancer cases or deaths estimated to have been caused by alcohol.

§ Alcohol-related cancers refer to all new cases or deaths for cancers whose risk is increased by alcohol consumption.

¶ In oesophagus cancer, only cases of squamous cell carcinoma were considered.
