# Supplementary material for: Modelling the impact of increased alcohol taxation on alcohol-attributable cancers in the WHO European Region
Source: Lancet Reg Health Eur. 2021 Sep 15;11:100225. doi: 10.1016/j.lanepe.2021.100225 (PMC8642705; doi:10.1016/j.lanepe.2021.100225)
Supplement: Supplementary file 2 [file mmc2.docx]

**Caption for supplementary material**

**Title:** Modelling the impact of increased alcohol taxation on alcohol-attributable cancers in the WHO European Region

**Authors:** Carolin Kilian, Pol Rovira, Maria Neufeld, Carina Ferreira-Borges, Harriet Rumgay, Isabelle Soerjomataram, Jürgen Rehm

Supplementary Table S1. Checklist of information (GATHER) that should be included in new reports of global health estimates.

Supplementary Table S2. Source excise duties and mean prices by country; data obtained Nov 2020.

Supplementary Table S3. Avoidable new alcohol-attributable cancer cases and deaths by cancer site for different tax-increase scenarios for the entire WHO European Region.

Supplementary Table S4. Avoidable new alcohol-attributable cancer cases and deaths for different tax-increase scenarios by countries of the WHO European Region.

Supplementary Table S5. Sensitivity analysis 1: Avoidable new alcohol-attributable cancer cases and deaths for applying the same tax rate as Finland, entire WHO European Region.

Supplementary Table S6. Sensitivity analysis 1: Avoidable new alcohol-attributable cancer cases and deaths for applying the same tax rate as Finland, by countries of the WHO European Region.

Supplementary Table S7. Sensitivity analysis 2: Avoidable new alcohol-attributable cancer cases and deaths for each tax increase scenario by cancer site and for the entire WHO European Region, applying a lag time between alcohol exposure and cancer development or deaths of 20 years.
